# Freshwater snails of biomedical importance in the Niger River Valley: evidence of temporal and spatial patterns in abundance, distribution and infection with *Schistosoma* spp.

**DOI:** 10.1186/s13071-019-3745-8

**Published:** 2019-10-22

**Authors:** Muriel Rabone, Joris Hendrik Wiethase, Fiona Allan, Anouk Nathalie Gouvras, Tom Pennance, Amina Amadou Hamidou, Bonnie Lee Webster, Rabiou Labbo, Aidan Mark Emery, Amadou Djirmay Garba, David Rollinson

**Affiliations:** 1 Department of Life Sciences, Natural History Museum, Cromwell Rd, South Kensington, London, SW7 5BD UK; 2 School of Biosciences, Cardiff University, Cardiff, CF10 3AT UK; 3 Réseau International Schistosomoses, Environnement Aménagement et Lutte (RISEAL-Niger), 333, Avenue des Zarmakoye, B.P. 13724, Niamey, Niger; 4 Centre de Recherche Médicale et Sanitaire (CERMES), Institut Pasteur International Network, 634 Bd de la Nation, BP 10887, Niamey, Niger; 5 World Health Organization, Geneva, Switzerland

**Keywords:** Schistosomiasis, Freshwater snails, Seasonality, Niger, *Bulinus*, *Biomphalaria*, *Schistosoma*, *B. truncatus*, *B. forskalii*, *B. pfeifferi*, *R. natalensis*, *S. haematobium*, *S. bovis*, *S. mansoni*

## Abstract

**Background:**

Sound knowledge of the abundance and distribution of intermediate host snails is key to understanding schistosomiasis transmission and to inform effective interventions in endemic areas.

**Methods:**

A longitudinal field survey of freshwater snails of biomedical importance was undertaken in the Niger River Valley (NRV) between July 2011 and January 2016, targeting *Bulinus* spp. and *Biomphalaria pfeifferi* (intermediate hosts of *Schistosoma* spp.), and *Radix natalensis* (intermediate host of *Fasciola* spp.). Monthly snail collections were carried out in 92 sites, near 20 localities endemic for *S. haematobium*. All bulinids and *Bi. pfeifferi* were inspected for infection with *Schistosoma* spp., and *R. natalensis* for infection with *Fasciola* spp.

**Results:**

*Bulinus truncatus* was the most abundant species found, followed by *Bulinus forskalii*, *R. natalensis* and *Bi. pfeifferi*. High abundance was associated with irrigation canals for all species with highest numbers of *Bulinus* spp. and *R. natalensis*. Seasonality in abundance was statistically significant in all species, with greater numbers associated with dry season months in the first half of the year. Both *B. truncatus* and *R. natalensis* showed a negative association with some wet season months, particularly August. Prevalences of *Schistosoma* spp. within snails across the entire study were as follows: *Bi. pfeifferi*: 3.45% (79/2290); *B. truncatus*: 0.8% (342/42,500); and *B. forskalii*: 0.2% (24/11,989). No *R. natalensis* (*n* = 2530) were infected. Seasonality of infection was evident for *B. truncatus*, with highest proportions shedding in the middle of the dry season and lowest in the rainy season, and month being a significant predictor of infection. *Bulinus* spp. and *Bi. pfeifferi* showed a significant correlation of snail abundance with the number of snails shedding. In *B. truncatus*, both prevalence of *Schistosoma* spp. infection, and abundance of shedding snails were significantly higher in pond habitats than in irrigation canals.

**Conclusions:**

Evidence of seasonality in both overall snail abundance and infection with *Schistosoma* spp. in *B. truncatus*, the main intermediate host in the region, has significant implications for monitoring and interrupting transmission of *Schistosoma* spp. in the NRV. Monthly longitudinal surveys, representing intensive sampling effort have provided the resolution needed to ascertain both temporal and spatial trends in this study. These data can inform planning of interventions and treatment within the region.

## Background

Schistosomiasis is a neglected tropical disease (NTD) affecting over 200 million people worldwide, with an at-risk population estimated at 700 million [1]. It is caused by digenean trematodes of the genus *Schistosoma.* The complex indirect life-cycle involves an intermediate freshwater snail host and transmission is through water contact. The distribution of schistosomes directly relates to the geographical range of their intermediate snail hosts. This is influenced by factors including climate, altitude, rainfall, water chemistry and aquatic vegetation [2]. Schistosomiasis is present throughout West Africa, including Niger. Urogenital schistosomiasis is endemic in the Niger River Valley (NRV), and is caused by S*chistosoma haematobium*, the most widespread and prevalent human schistosome species across Africa, which displays often severe pathologies [3–6]. *Schistosoma bovis*, a pathogen of domestic livestock and some non-domestic artiodactyls [7] is also prevalent in the region [8–11]. *Schistosoma haematobium* and *S. bovis* have overlapping distributions across mainland Africa [12], and can infect several different species of the freshwater snail genus *Bulinus* as their intermediate hosts [13–15]. These two species of schistosome show evidence of hybridization in several West African countries, including Niger, complicating disease control [16–20]. In addition, the NRV has localised areas of intestinal schistosomiasis [21], which appear to be spreading (A. Garba, personal communication). Intestinal schistosomiasis is caused by *Schistosoma mansoni,* which infects over 83 million people across sub-Saharan Africa, the Middle East, parts of South America and some Caribbean islands [22, 23]; snail species of the genus *Biomphalaria* act as the intermediate host [24].

Together, the multiple snail and schistosome species result in a complex and persistent pattern of schistosomiasis transmission in Niger, influenced by the country’s geography. The Niger River, which crosses approximately 550 km of western Niger, is the country’s main water supply, critical in a country which is two-thirds desert [25]. The catchment is home to freshwater snail species of biomedical importance, including the pulmonate snails *Bulinus truncatus*, *B. globosus*, *B. senegalensis*, *B. forskalii* and *Biomphalaria pfeifferi*, all acting as hosts for *Schistosoma* spp., and *Radix natalensis*, a host for *Fasciola* spp. [24]. Studies have been undertaken in West Africa on how dam building has impacted schistosomiasis distributions by altering available habitat for freshwater snail intermediate hosts [23]. Some recent studies have shed light on abundance and distribution of these snail species at different spatial and temporal scales, both in the NRV and in sub-Saharan Africa as a whole [26–32]. However, substantial knowledge gaps remain. In addition, there is often a mismatch between intermediate host snail abundance and distribution and snail infection, let alone between snail infection levels and human schistosomiasis transmission [33–36]. For example, analysis of a recent outbreak of urogenital schistosomiasis in Corsica found evidence of ongoing transmission but no infected Bulinid snails [37]. It is not clear if this is due to insufficient sampling, the characteristic low levels of infection (and latent infection period) in snails, or if transmission is so patchy that there is little correlation of snail abundance and infection prevalence, and resulting transmission [38]. Schistosomiasis is highly focal, requiring overlap of intermediate and definitive hosts [24], and multiplication of the larval cercaria stage in snails can continue transmission even with very low snail infection prevalence [37]. Recent reviews therefore highlight the crucial importance of snail surveys to aid understanding of transmission, and to improve predictive modelling of future schistosomiasis distributions in relation to climate change and schistosomiasis control [39–41]. Currently there is a focus on longitudinal survey data across years, and critically, seasons, to characterize snail populations with more precision [42]. These surveys require accurate identification of snails and schistosome cercariae to provide high quality data to support treatment program activities and contribute to schistosomiasis knowledge more widely. The Schistosomiasis Consortium for Operational Research and Evaluation programme (SCORE, https://score.uga.edu) has recently undertaken studies to investigate and quantify the factors related to snail-human infection processes within the context of mass drug administration strategies in five African countries, including Niger [43]. Among other study goals, the programme aimed to address the gap in longitudinal abundance and distribution data for intermediate freshwater snail hosts to help inform public health planning for schistosomiasis control within Niger. Here, we report on longitudinal surveys, carried out in the context of the SCORE studies, for several species of freshwater snails of biomedical importance in the Niger River Valley. Specifically, this study conducted surveys to identify *Schistosoma* spp. transmission sites and to determine the intermediate snail hosts (particularly *Bulinus* spp.) at these sites and the parameters that influence intermediate snail host abundance and disease transmission potential.

## Methods

### Study region

The Niger River Valley has a Sahelian ecotone and climate. Highly seasonal, the first half of the year has very low or no rainfall, but flooding is frequent during the rainy season in the second half of the year. The study area transects different (broad) ecological regions, the Bassin des Dallois on the east side of the river, (a relatively more productive zone), the Liptako Sahel on the northwest side and the Plateau Goumantche on the southwest [25]. Other notable features of the study region are extensive rice growing areas supported by irrigation canal systems along the river, and the Kandadji Dam north of Tillaberi, which has been under construction since 2008 [21, 44].

### Surveys

**Fig. 1:**
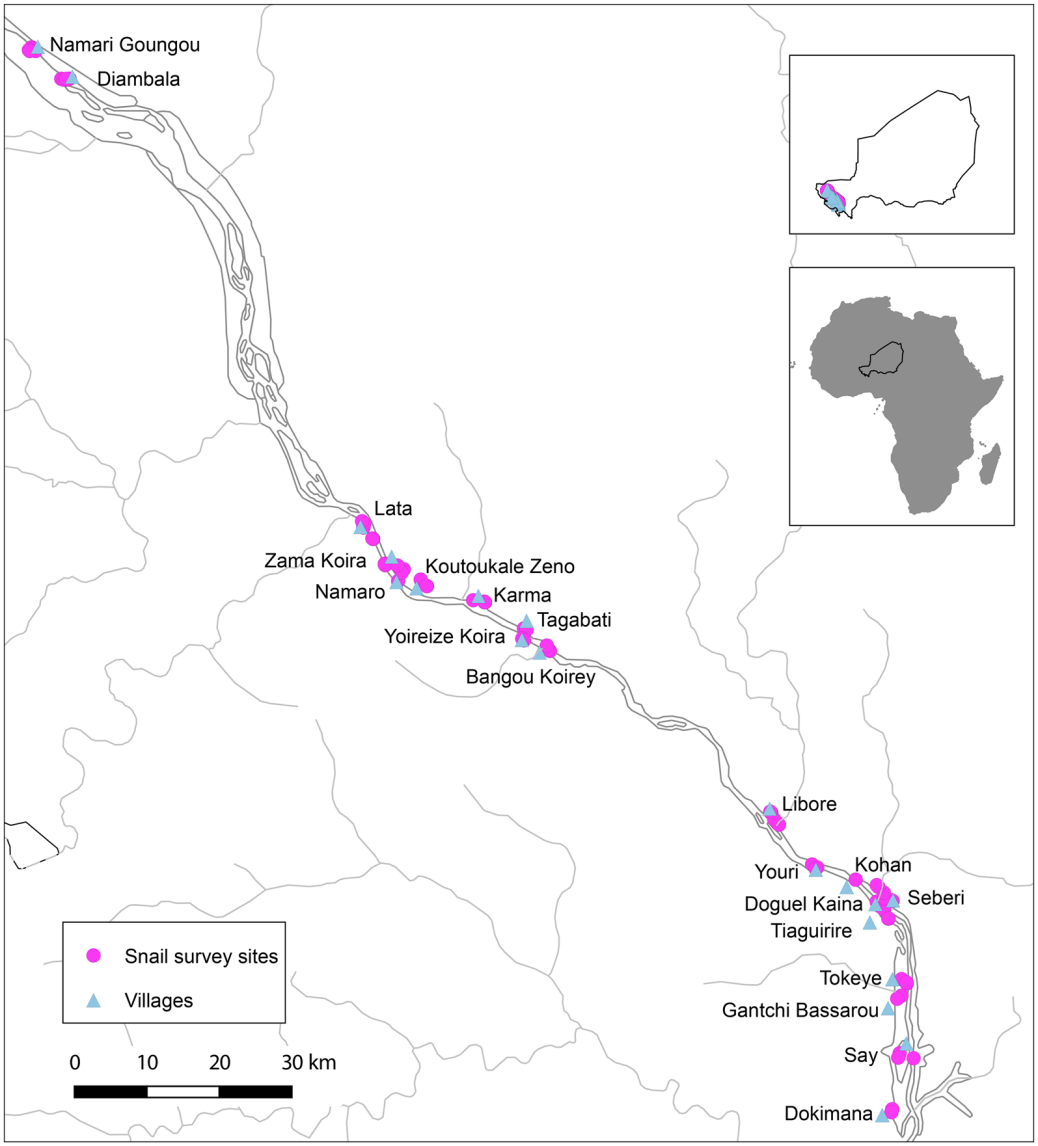
Map of sampling localities showing locations of snail survey sites and villages in the study

**Fig. 2:**
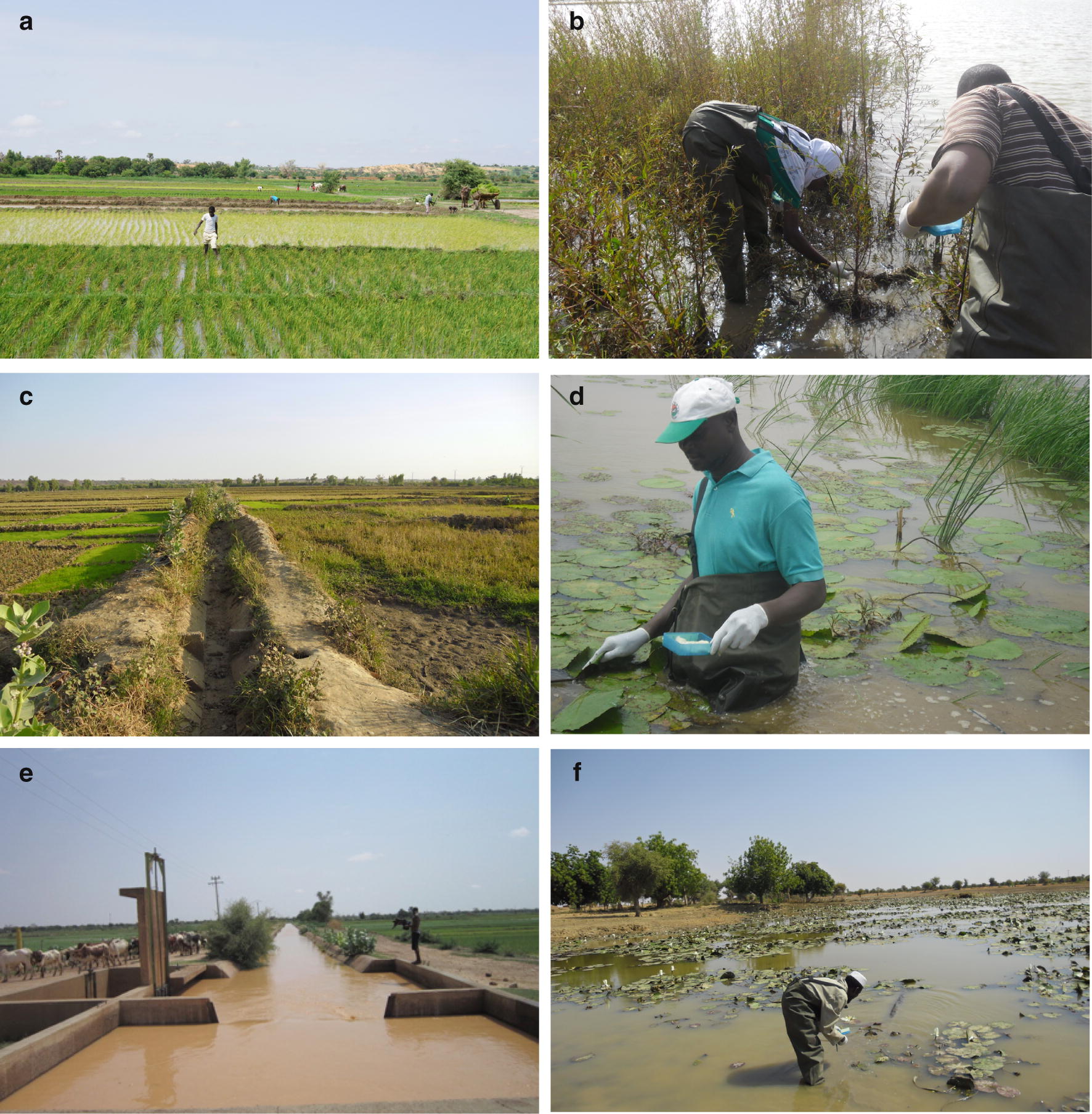
Examples of some of the site types surveyed. **a** Rice paddy. **b** River. **c** Tertiary irrigation canal. **d** Pond. **e** Secondary irrigation canal. **f** Spillway. Photo credit Amadou Garba

Monthly snail surveys were undertaken between July 2011 and January 2016 in 92 potential transmission sites, near 20 villages associated with human schistosomiasis (Table 1, Additional file 1: Table S1). Sites were surveyed every month (apart from April in 2014 and 2015 due to logistical reasons). Site selection was based on local knowledge of water contact sites and snail presence. Altogether, 16 villages were from the wider SCORE programme and four from an earlier study, CONTRAST [45], including two northern villages, Namari Goungou and Diambala, mixed infection foci with evidence of *S. mansoni* [44, 46]. Villages here are written as localities as additional villages not included in the study are often close by. Most study sites are within approximately 60 km up or downstream of Niamey, apart from these two northerly localities which are approximately double that distance from Niamey (Fig. 1). The survey covered a range of site types including irrigation canals, both concrete-lined secondary canals, which draw water directly from the river, and the smaller dirt-lined tertiary canals, branching off secondary canals to deliver water to rice paddies; the rice paddies themselves, the river (shallows of the main body of the Niger River), rivulets (small streams), ponds, and spillways (floodplain of tributaries feeding into the river) (Fig. 2). One stream site (at Say) was also surveyed but as it was the only one of its type, was excluded from final analysis. Irrigation canals were the most sampled sites, because of prior knowledge of high *Bulinus* spp. densities there from the earlier CONTRAST studies. Snail species surveyed were from the genera *Bulinus* and *Biomphalaria* (both Planorbidae), and *Radix* (Lymnaeidae). Species included *Bulinus truncatus* and *B. globosus*, which have a degree of morphological overlap [47], *B. forskalii* and *B. senegalensis* (again with some morphological overlap), *Biomphalaria pfeifferi* and *Radix natalensis*. Sites were examined for snails by two collectors, scooping and examining vegetation for approximately 15-min intervals per site. If abundance was very low, the interval was doubled to 30 min. At each site, GPS was recorded on a handheld Global Positioning System GPS (Garmin eTrex, Taiwan), and water chemistry including pH, total dissolved solids (TDS), and conductivity were recorded during site visits (apart from 2015 due to equipment failure) on a handheld water meter (Thermo Scientific Eutech Multiparameter PCTEST35K, Fisher Scientific UK Ltd., Loughborough, UK). Other relevant parameters such as water temperature, estimated water flow and depth were also recorded. All collected snails were taken back to the laboratory and morphologically identified to the species level, and the number of each species per site was recorded. Weather station data were acquired from the Niamey Weather Station (ID 61052), and downloaded from the USDA Foreign Agricultural Service weather meteorological office (WMO) pecad site in October, 2018 (https://gis.pecad.fas.usda.gov/WmoStationExplorer/).

**Table 1:** Snail survey site list, showing totals of sites by site type and locality/village

District	Locality	Canal 2	Canal 3	Pond	Rice	River	Rivulet	Spillway	*n*
Kollo	Bangou Koirey						3		3
Tillaberi	Diambala	3	4						7
Say	Doguel Kaina	1	1		1	1	1		5
Say	Dokimana			2					2
Say	Gantchi Bassarou			1	1				2
Kollo	Karma	1	2		1	1	1		6
Say	Kohan Garantche					1			1
Say	Koutoukale Zeno	2	2	1					5
Kollo	Lata Kabia	3	2	1	1			1	8
Kollo	Libore	6	2		2				10
Tillaberi	Namari Goungou	2	4						6
Kollo	Namaro	1	1	2		1			5
Say	Say	1				1		1	3
Kollo	Seberi	4	2						6
Kollo	Tagabati					2	1		3
Kollo	Tiaguirire	1	1		1				3
Kollo	Tokeye	2	1	1					4
Kollo	Yoreize Koira	1	2		1				4
Kollo	Youri				1	1	1		3
Kollo	Zama Koira Tegui	2	2		1			1	6
		30	26	8	10	8	7	3	92

*Notes*: Canal 2, secondary irrigation canal; Canal 3, tertiary irrigation canal; rice, rice paddy; n, total number of sites for a given locality/village

### Checking for snail infection status

All snails were individually placed in clean freshwater in a well within a 12-well microtiter plate, exposed to light and checked for the shedding of cercariae at different intervals; first, one to three days after collection, and additionally at two weeks to one month post-collection [2]. Snails were kept in the wells for several hours while shedding was induced, then placed in aquaria for the interim period between shedding attempts. Snails were inspected under a stereo-microscope for shedding of schistosome cercariae, which were identified by using a key [48]. If shedding *Schistosoma* spp. cercariae, snails were recorded as positive for patent infection (hereafter, referred to as ‘shedding’). The cercariae were individually collected by micro-pipette in 3–5 µl of water and preserved on Whatman FTA cards (GE Healthcare Life Sciences, Buckinghamshire, UK) for any future molecular identification [49]. All snails were preserved in 100% ethanol to allow future molecular analysis. Both the snail voucher specimens and cercariae were archived with their contextual data in the Schistosomiasis Collection at the Natural History Museum, SCAN [50].

### Statistical analysis

All data analysis was undertaken in R version 3.5.3 “Great Truth” [51] and R-Studio [52]. All statistical tests were conducted with the significance level α = 0.05 for rejecting null hypotheses. Exploratory data analysis was performed to identify broad scale spatial and/or temporal trends, and test statistical assumptions, following Zuur et al. [53]. This revealed high collinearity between water conductivity and TDS, and we proceeded to include only the former variable in the following models.

Variation in snail counts was analysed using generalized linear mixed effect models (GLMM) in the package *glmmTMB* [54]. This package efficiently fits negative binomial models that can account for overdispersed count data, while also allowing for arguments to account for zero inflation, if needed [55]. The fit of all constructed models was investigated visually and statistically using a simulation-based approach in the package *DHARMa* [56]. Models with good fit showed no significant deviation in the QQ plot of simulated residuals, and passed a non-parametric dispersion test (function ‘testDispersion()’). In all count models, a negative binomial distribution showed an improved fit over the Poisson distribution, and was therefore chosen as the family. Zero inflation, which assumes a mix of structural and sampling zero data, was initially considered as initial data exploration revealed a high proportion of zeros in the data, which can indicate zero inflation, or overdispersion relative to the Poisson distribution [55]. However, we did not find sufficient reason to consider zeros as structural, and zero inflation tests in *DHARMa* (function: ‘testZeroInflation()’) did not show statistical support that data were zero-inflated. We therefore did not include terms for zero inflation in the models. Since sampling sites were not spatially independent and could show variation in intercepts due to varying initial snail abundance, we included sites as a random intercept term for count models, nested in locality to reflect the sampling structure. To account for the temporal pseudoreplication caused by repeatedly measuring over time, we included the collection date as a random intercept term. Sampling duration varied, and was therefore included as an offset in the model. Fixed main effects were included if they were ecologically meaningful potential influences on snail abundances, totals of shedding snails, or prevalence of *Schistosoma* spp. infection within the snails (proportion of snails shedding *Schistosoma* spp. as a percentage of the total). We did not conduct stepwise single term deletion on the maximal model, due to the issues associated with model simplification [57, 58]. Models investigating overall *Bulinus* spp. counts included water temperature, pH, water speed, water depth, water conductivity, precipitation, locality, site type and total number of shedding *Bulinus* spp. snails as fixed main effects, with interactions between site type and water temperature, pH, conductivity, and precipitation respectively. Models investigating counts on the species level of a given species included precipitation, site type, month, locality and total counts of the remaining species of snails as fixed main effects. Continuous variables were centred and scaled to the mean. Models investigating counts of shedding snails for *B. truncatus*, *B. forskalii* and *Bi. pfeifferi* included site type, month and locality as fixed main effects.

Variation in *Schistosoma* spp. prevalence in the snail hosts studied was analysed using simple generalized linear models, based on abundance data pooled by month. As here we were modelling proportional data, we chose a binomial distribution, with prevalence specified as the number of shedding snails divided by total snails per locality/timepoint, weighted in the model by the total number of snails. Significance of terms in all models were retrieved using the function ‘Anova.glmmTMB()’ for glmmTMB models, and function ‘Anova()’ from the package *car* [59] for glmm models. For models of shedding *B. forskalii*, terms were run separately because of convergence problems owing to small sample size. For some models also, localities with zero abundance were removed. For statistically significant terms, we conducted *post-hoc* tests using the *emmeans* package [60].

## Results

**Fig. 3:**
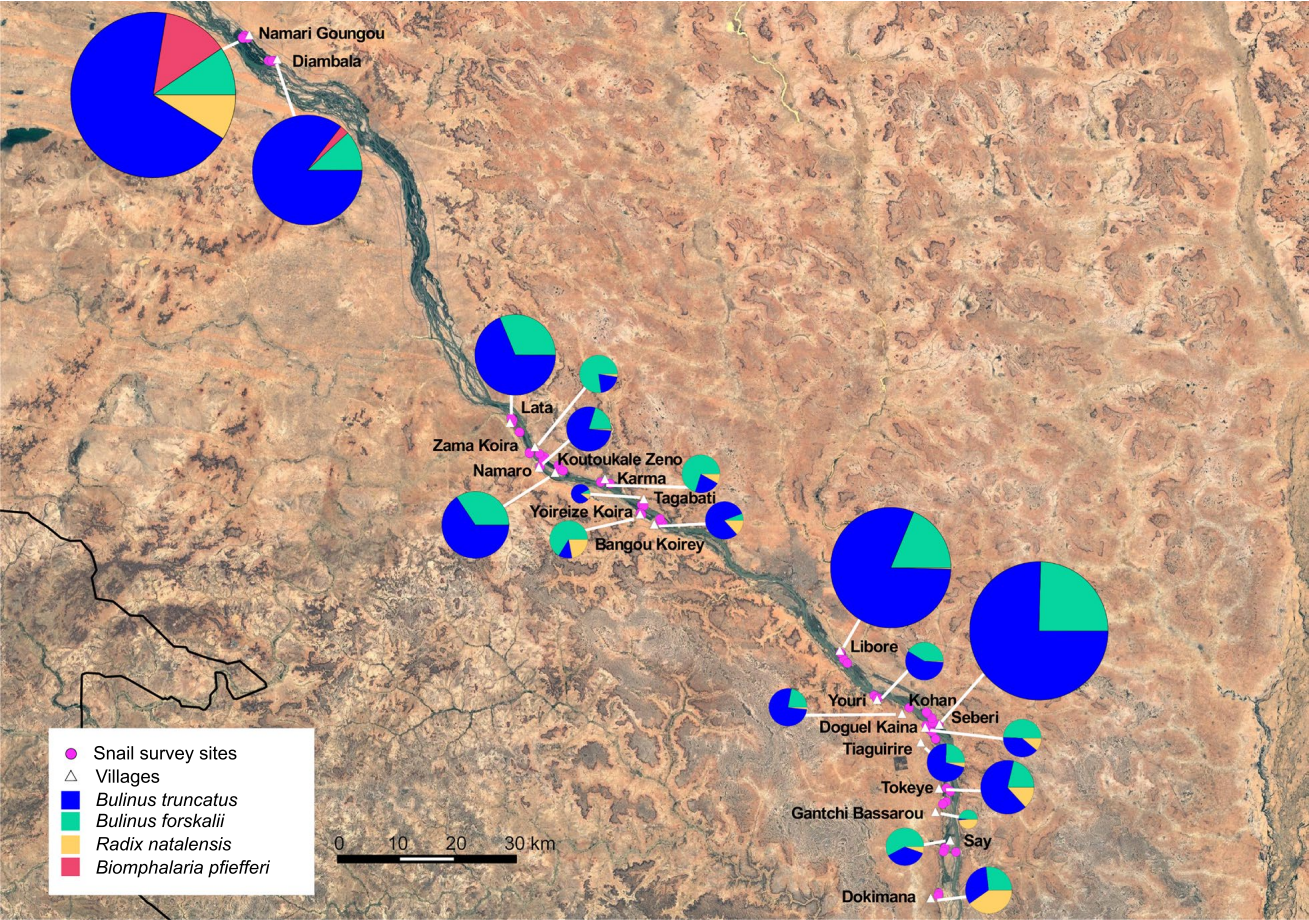
Snail species by locality, pie charts scaled by proportion of total snails found. Data shown: collected field data (final dataset for analysis), not modelled counts. Apart from Koutoukale Zeno village, *R. natalensis* was found at all localities, although was evident in very low numbers at several localities, e.g. Seberi

A total of 59,674 snails were found throughout the four and a half year study. *Bulinus truncatus* was the most abundant (*n* = 42,500), followed by *B. forskalii* (*n* = 11,989), *R. natalensis* (*n* = 2530) and *Bi. pfeifferi* (*n* = 2290) (Fig. 3, Table 2). *Bulinus globosus* and *B. senegalensis* were also present but found in low numbers (*n* = 290 and *n* = 76 in total, respectively, Table 2). Prevalences of *Schistosoma* spp. within snails across the entire study were as follows: *Bi. pfeifferi*: 3.45% (79/2290); *B. truncatus*: 0.8% (342/42,500); and *B. forskalii*: 0.2% (24/11,989). No *R. natalensis* (*n* = 2530) were infected.

**Table 2:** Snail survey species data broken down by locality, site type and month

	*n*	Bul.r	Bul	Bul+	BT.r	BT	BT+	BT.p	BF.r	BF	BF+	BF.p
Locality												
Bangou Koirey	55	8.2	453	10	7.4	406	10	2.46	0.6	31	0	0.00
Diambala	250	30.1	7529	32	26.0	6506	32	0.49	3.7	914	0	0.00
Doguel Kaina	134	5.3	704	6	2.2	296	6	2.03	3.0	397	0	0.00
Dokimana	50	12.1	606	14	5.8	290	13	4.48	6.3	316	1	0.32
Gantchi Bassarou	59	1.3	75	5	0.1	3	0	0.00	1.2	72	5	6.94
Karma	87	6.2	542	2	1.5	128	2	1.56	4.7	409	0	0.00
Kohan Garantche	35	7.3	254	0	5.1	179	0	0.00	2.1	75	0	0.00
Koutoukale Zeno	130	20.8	2708	5	13.8	1789	5	0.28	6.7	867	0	0.00
Lata Kabia	179	22.6	4046	10	15.6	2787	9	0.32	7.0	1259	1	0.08
Libore	270	30.8	8320	150	24.8	6694	143	2.14	5.8	1565	7	0.45
Namari Goungou	233	54.1	12,594	11	47.2	10,987	9	0.08	6.6	1530	1	0.07
Namaro	99	10.2	1009	3	8.1	798	2	0.25	2.1	211	1	0.47
Say	94	7.3	683	1	2.7	258	1	0.39	4.3	402	0	0.00
Seberi	255	43.6	11,123	46	35.0	8929	43	0.48	8.6	2194	3	0.14
Tagabati	52	3.0	155	0	2.7	142	0	0.00	0.3	13	0	0.00
Tiaguirire	69	9.7	670	0	7.2	496	0	0.00	2.4	163	0	0.00
Tokoye	112	13.1	1470	60	10.0	1117	57	5.10	3.2	354	3	0.85
Yoreize Koira	102	4.3	437	0	0.6	66	0	0.00	3.6	371	0	0.00
Youri	99	8.6	850	3	5.0	497	2	0.40	3.6	352	1	0.28
Zama Koira Tegui	132	4.7	626	9	1.0	132	8	6.06	3.7	494	1	0.20
Total	2496	22	54,854	367	17.0	42,500	342	0.80	4.8	11,989	24	0.20
Site type												
Canal 2	924	30.4	28,094	137	25.3	23,362	131	0.56	5.0	4590	5	0.11
Canal 3	715	28.5	20,349	114	22.0	15,715	112	0.71	6.3	4518	2	0.04
Pond	209	11.6	2422	81	6.4	1347	71	5.27	4.9	1024	10	0.98
Rice	215	7.7	1659	14	2.7	571	7	1.23	5.0	1078	7	0.65
River	208	5.3	1105	4	3.9	815	4	0.49	1.4	284	0	0.00
Rivulet	141	5.0	705	16	4.4	624	16	2.56	0.5	64	0	0.00
Spillway	84	6.2	520	1	0.8	66	1	1.52	5.1	431	0	0.00
Total	2496	22	54,854	367	17.0	42,500	342	0.80	4.8	11,989	24	0.20
Month												
January	232	17.0	3951	17	14.7	3405	16	0.47	2.3	536	1	0.19
February	224	27.5	6157	66	21.9	4895	64	1.31	5.6	1261	2	0.16
March	216	47.4	10,242	109	38.6	8330	100	1.20	8.9	1913	9	0.47
April	41	56.2	2305	20	45.9	1882	20	1.06	10.3	423	0	0.00
May	185	30.6	5652	21	24.4	4522	20	0.44	6.1	1130	1	0.09
June	173	32.3	5592	52	26.0	4502	51	1.13	5.2	895	1	0.11
July	227	17.1	3887	22	14.2	3221	18	0.56	2.6	595	4	0.67
August	197	12.0	2355	4	7.8	1543	3	0.19	4.0	797	1	0.13
September	215	17.6	3782	8	9.4	2026	6	0.30	7.9	1704	2	0.12
October	279	12.7	3551	18	8.7	2433	16	0.66	4.0	1118	1	0.09
November	232	15.7	3648	11	12.1	2810	11	0.39	3.6	838	0	0.00
December	275	13.6	3732	19	10.7	2931	17	0.58	2.8	779	2	0.26
Total	2496	22	54,854	367	17.0	42,500	342	0.80	4.8	11,989	24	0.20

	BG	BS	BP.r	BP	BP+	BP.p	RN.r	RN
Locality								
Bangou Koirey	16	0	0.0	0	0		1.3	70
Diambala	109	0	0.8	210	3	1.43	0.0	7
Doguel Kaina	11	0	0.0	0	0		0.6	78
Dokimana	0	0	0.0	0	0		7.3	365
Gantchi Bassarou	0	0	0.0	0	0		1.1	63
Karma	5	0	0.0	0	0		0.6	51
Kohan Garantche	0	0	0.0	0	0		0.1	3
Koutoukale Zeno	0	52	0.0	0	0		0.0	0
Lata Kabia	0	0	0.0	0	0		0.0	6
Libore	61	0	0.0	0	0		0.1	32
Namari Goungou	77	0	8.9	2080	76	3.65	6.1	1432
Namaro	0	0	0.0	0	0		0.1	12
Say	0	23	0.0	0	0		0.0	1
Seberi	0	0	0.0	0	0		0.0	1
Tagabati	0	0	0.0	0	0		0.3	15
Tiaguirire	11	0	0.0	0	0		0.3	23
Tokoye	0	0	0.0	0	0		2.0	225
Yoreize Koira	0	0	0.0	0	0		1.2	124
Youri	0	1	0.0	0	0		0.1	6
Zama Koira Tegui	0	0	0.0	0	0		0.1	16
Total	290	76	0.9	2290	79	3.45	1.0	2530
Site type								
Canal 2	142	0	2.0	1819	46	2.53	0.1	53
Canal 3	116	0	0.7	471	33	7.01	2.2	1541
Pond	0	52	0.0	0	0		2.9	610
Rice	10	0	0.0	0	0		0.8	168
River	5	1	0.0	0	0		0.3	69
Rivulet	17	0	0.0	0	0		0.6	88
Spillway	0	23	0.0	0	0		0.0	1
Total	290	76	0.9	2290	79	3.45	1.0	2530
Month								
January	10	0	1.1	262	10	3.82	0.6	134
February	0	1	2.9	656	13	1.98	2.5	554
March	0	0	3.0	646	10	1.55	4.0	870
April	0	0	2.0	80	5	6.25	9.0	368
May	0	0	0.8	154	5	3.25	0.8	156
June	195	0	0.6	102	9	8.82	0.8	137
July	0	71	0.1	15	2	13.33	0.3	74
August	11	4	0.1	16	0	0.00	0.1	23
September	52	0	0.4	78	2	2.56	0.1	15
October	0	0	0.1	25	2	8.00	0.1	20
November	0	0	0.9	206	17	8.25	0.1	25
December	22	0	0.2	50	4	8.00	0.6	154
Total	290	76	0.9	2290	79	3.45	1.0	2530

*Abbreviations*: n, total count i.e. total number of site visits per category (e.g. 84 records in total from site visits to spillway sites); Bul.r, total count divided by n; Bul, *Bulinus* spp. raw count; Bul*+*, *Bulinus* spp. total shedding i.e. positive for *Schistosoma* spp. infection; BT.r, *B. truncatus* count divided by n; BT, *B. truncatus* raw count, BT+, *B. truncatus* total positive for *Schistosoma* spp. infection; BT.p, *B. truncatus* prevalence; BF.r, *B. forskalii* count divided by n; BF, *B. forskalii* raw count; BF+, *B. forskalii* total positive for *Schistosoma* spp. infection; BF.p, *B. forskalii* prevalence; BG, *B. globosus*; BS, *B. senegalensis*; BP.r, *Bi. pfeifferi* total count divided by n; BP, *Bi. pfeifferi* raw count; BP+, *Bi. pfeifferi* positive for *Schistosoma* spp. infection; BP.p, *Bi. pfeifferi* prevalence; RN, *R. natalensis*; RN.r, total count divided by n

### Effect of locality on snail abundance

**Fig. 4:**
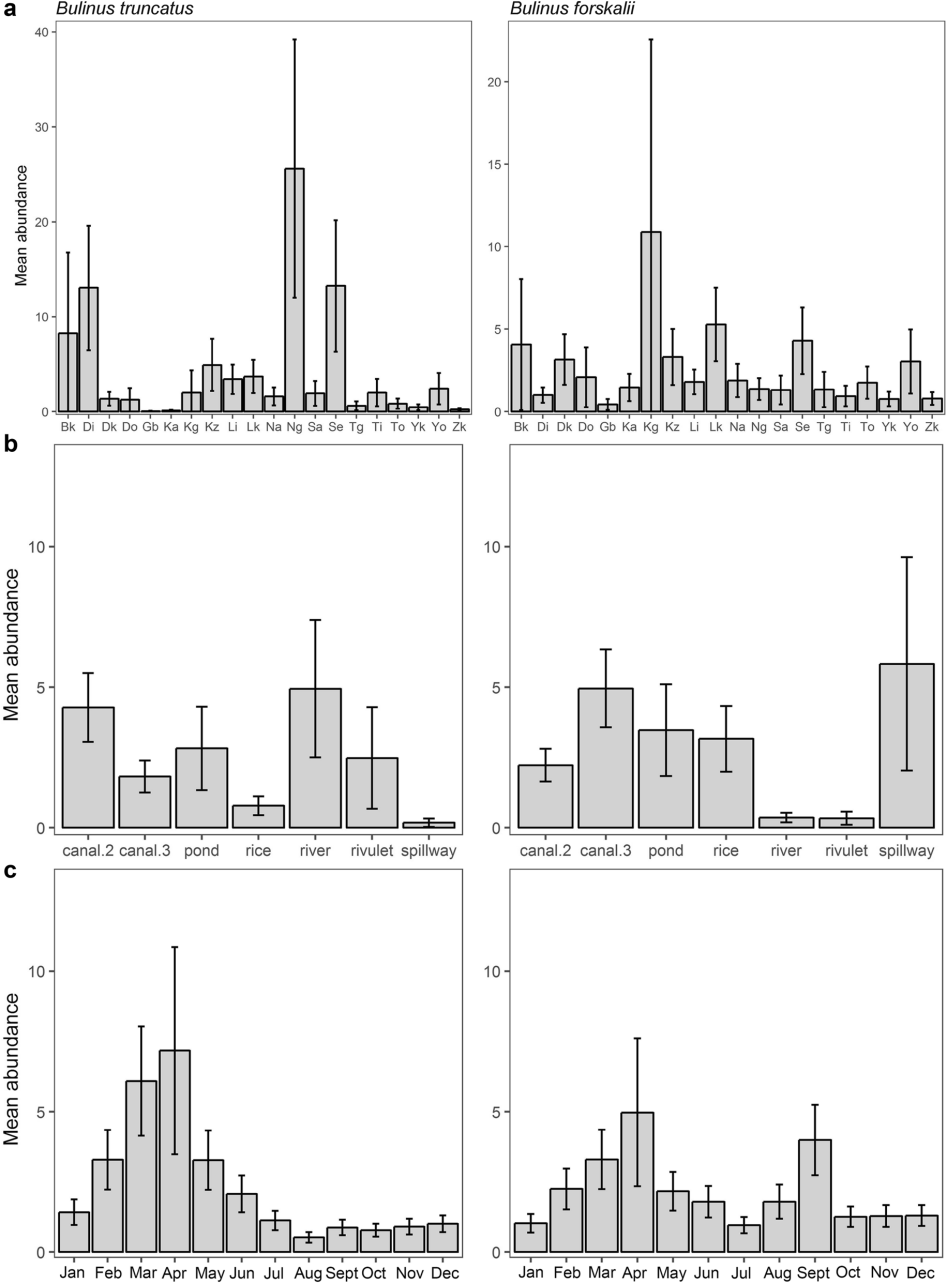
**a** *Bulinus truncatus* and *B. forskalii* modelled mean abundance by locality, a significant predictor of abundance for both species: *B. truncatus* (*χ*^2^ = 107, *df* = 18, *P* < 0.001); *B. forskalii* (*χ*^2^ = 34, *df* = 19, *P* < 0.001). **b** *B. truncatus* and *B. forskalii* modelled mean abundance by site type, a significant predictor of abundance for both species: *B. truncatus* (*χ*^2^ = 33.2, *df* = 6, *P* < 0.001); *B. forskalii* (*χ*^2^ = 27.8, *df* = 6, *P* < 0.001). **c** *B. truncatus* and *B. forskalii* modelled mean abundance by month, a significant predictor of abundance for both species: *B. truncatus* (*χ*^2^ = 85.4, *df* = 11, *P* < 0.001); *B. forskalii* (*χ*^2^ = 32.4, *df* = 11, *P* < 0.001. *Bk* Bangou Koirey, *Di* Diambala, *Dk* Doguel Kaina, *Do* Dokimana, *Gb* Gantchi Bassarou, *Ka* Karma, *Kg* Kohan Garantche, *Kz* Koutoukale Zeno, *Li* Libore, *Lk* Lata Kabia, *Na* Namaro, *Ng* Namari Goungou, *Sa* Say, *Se* Seberi, *i* Tagabati, *Ti* Tiaguirire, *To* Tokoye, *I* Yoreize Koira, *Yo* Youri, *Zk* Zama Koira Tegui, *canal.2* secondary irrigation canal, *canal.3* tertiary irrigation canal; *rice* rice paddy

Differences in abundances across localities was evident in all species surveyed (Fig. 3). *Bulinus truncatus*, most abundant at the majority of localities, showed an almost bimodal distribution of either very low abundances or relatively much higher numbers at a few localities: Namari Goungou, Seberi and Diambala (Fig. 4a). Locality was a highly significant predictor of abundance in *B. truncatus* (*χ*^2^ = 107, *df* = 18, *P* < 0.001, Table 3). *Bulinus forskalii* was found in lower numbers than *B. truncatus*, with a less variable distribution, but the differences across localities were still significant (*χ*^2^ = 34, *df* = 19, *P* < 0.001, Table 3). Seven localities had higher abundances of *B. forskalii* than *B. truncatus*, all with low numbers overall (Fig. 4a, Table 2). Locality was also a significant predictor of abundance in *R. natalensis* (*χ*^2^ = 46.4, *df* = 19, *P* < 0.001, Table 4). This species had low abundances overall, and was found in very low numbers at several sites and absent altogether from Koutoukale Zeno (Fig. 3). *Biomphalaria pfeifferi* was found only at Namari Goungou and Diambala, with higher abundance at the former; however, this difference was not significant (*P* = 0.09).

**Table 3:** Summary statistics of all glmmTMB models for *B. truncatus* and *B. forskalii* abundance, reporting *χ*^2^ *df* and *P*-values, all negative binomial apart from binomial for prevalence models

Term	*B. truncatus*			BT positive			BT prevalence			*B. forskalii*			BF positive			BF prevalence		
	*χ* ^2^	*df*	*P*	*χ* ^2^	*df*	*P*	*χ* ^2^	*df*	*P*	*χ* ^2^	*df*	*P*	*χ* ^2^	*df*	*P*	*χ* ^2^	*df*	*P*
Precipitation	0.4	1	0.548							0.6	1	0.427						
Site type	33.2	6	< 0.0001	15	6	0.02	92.9	6	< 0.0001	27.8	6	< 0.0001	4.3	7	0.742	9.5	7	0.219
Locality	107	18	< 0.0001	41.7	19	0.002	139.4	13	< 0.0001	34	19	0.018	2	19	1	13.5	9	0.139
Month	85.4	11	< 0.0001	62.3	11	0	18.9	11	0.063	32.4	11	0.001	0.1	1	0.781	29.4	11	0.002
BT positive total	33.8	1	< 0.0001															
BF positive total										2.4	1	0.119						

*Key*: Headers: names for given model, BT/BF positive: model of abundance of shedding snails for *B. truncatus*, *B. forskalii* and *Bi. pfeifferi*, respectively; BT/BF prevalence: model of *Schistosoma* spp. prevalence (proportion of snails shedding) in the given species; precipitation, WMO weather station precipitation data

**Table 4:** Summary statistics of all glmmTMB models for *B. pfeifferi* and *R. natalensis*, reporting *χ*^2^, *df* and *P*-values, all negative binomial apart from binomial for prevalence model

Term	*Bi. pfeifferi*			BP positive			BP prevalence			*R. natalensis*		
	*χ* ^2^	*df*	*P*	*χ* ^2^	*df*	*P*	*χ* ^2^	*df*	*P*	*χ* ^2^	*df*	*P*
Precipitation	0.4	1	0.527							0.1	1	0.787
Site type	3.1	1	0.079	0.5	1	0.486	7.1	1	0.008	15.7	6	0.016
Locality	3	1	0.086	8.2	1	0.004	5.1	1	0.023	46.4	19	< 0.0001
Month	20.9	11	0.035	1	1	0.315	24.3	9	0.004	122	11	< 0.0001
BP positive total	19.1	1	< 0.0001									

*Key*: BP positive: model of abundance of shedding snails for *Bi. pfeifferi*; BP prevalence: model of *Schistosoma* spp. prevalence

### Effect of site type on snail abundance

*Bulinus truncatus* was significantly more abundant in secondary than tertiary canals, and had lowest abundance in spillways and rice paddies. Site type was a significant predictor of abundance in *B. truncatus* (*χ*^2^ = 33.2, *df* = 6, *P* < 0.001, Fig. 4b, Additional file 1: Figure S1). In contrast, *B. forskalii* was more abundant in tertiary than secondary canals, and also had high abundance in pond, rice paddy and spillway sites (although the latter show very large standard errors due to large variation). River and rivulet sites had very low abundances. These differences were also significant (*χ*^2^ = 27.8, *df* = 6, *P* < 0.001). *Radix natalensis* were mostly found in tertiary canals, then ponds, with very low numbers in secondary canals, contrasting with both *Bulinus* species and *Bi. pfeifferi.* Site type was also a significant predictor of abundance (*χ*^2^ = 15.7, *df* = 6, *P* = 0.016). *Biomphalaria pfeifferi* was only found in tertiary and secondary canals, particularly the latter, but the difference was not significant (*P* = 0.08).

### Effect of month on snail abundance

All species surveyed showed significant seasonality in abundance, particularly *B. truncatus* and *R. natalensis*. *B. truncatus* abundance had a significant positive association with dry season months February to May, and a significant negative association with the wet season month of August (Fig. 4c, Additional file 1: Table S2; note that large standard errors are evident for April because surveys were not undertaken for this month in 2014 and 2015). Month was a significant predictor of *B. truncatus* abundance (*χ*^2^ = 85.4, *df* = 11, *P* < 0.001). For the first half of the year, *B. forskalii* showed similar patterns to *B. truncatus*; however, later in the year there was an additional peak in abundance (Fig. 4c). *Bulinus forskalii* abundance showed a significant positive association with February through to May, and also the wet season month of September (Fig. 4c, Additional file 1: Table S3). Month was a significant predictor of *B. forskalii* abundance (*χ*^2^ = 32.4, *df* = 11, *P* = 0.001). *Radix natalensis* abundance showed significant positive associations with February through April, and a significant negative association for July to October (Additional file 1: Table S4). *Radix natalensis* had marked significant seasonal variation overall (*χ*^2^ = 121.7, *df* = 11, *P* < 0.001). In *Bi. pfeifferi*, although counts were more variable by month than either for *R. natalensis* or *Bulinus* spp., month was also a significant predictor of abundance (*χ*^2^ = 20.9, *df* = 11, *P* = 0.035, Additional file 1: Table S5).

### Effect of water variables on snail abundance

Although significant seasonality on abundance in all species surveyed appeared correlated with precipitation, the effect of precipitation on snail abundance was not significant for either *Bulinus* spp. pooled or testing any species separately. However, there was a marginally significant interaction of precipitation and site type for *Bulinus* spp. abundance (*χ*^2^ = 18.7, *df* = 6, *P* = 0.05, Table 5), with a significant positive association for pond and rice paddy sites and negative for rivulets (Additional file 1: Table S6). A significant interaction of water temperature and site type with *Bulinus* spp. abundance was also evident (*χ*^2^ = 31.1, *df* = 6, *P* < 0.001), with significant negative associations for river and rivulet sites, and a significant positive association for spillways (i.e. higher temperatures in river and rivulet sites were associated with higher numbers of *Bulinus*, and *vice versa* for spillway; Additional file 1: Figure S2). An interaction of conductivity and site type with *Bulinus* abundance was significant (*χ*^2^ = 21, *df* = 6, *P* = 0.002), with ponds showing a significant positive association, and rivulets a negative association (Additional file 1: Table S6). Considering single terms, water speed had a significant negative association with *Bulinus* spp. abundance (*χ*^2^ = 14.6, *df* = 1, *P* < 0.001, Additional file 1: Figure S3), as did water depth (*χ*^2^ = 6.7, *df* = 1, *P* =  0.01). All water variable data are summarised in Additional file 1: Table S7.

**Table 5:** Summary statistics of glmmTMB (negative binomial) model for *Bulinus* spp., reporting *χ*^2^, *df* and *P*-values

	*χ* ^2^	*df*	*P*
Water temperature	0.4	1	0.552
pH	0.6	1	0.445
Water speed (m/s)	14.6	1	0.000
Water depth (cm)	6.7	1	0.010
Conductivity	5.6	1	0.018
Precipitation	1.3	1	0.252
Site type	9.9	6	0.131
*Bulinus* positive tot	30.6	1	< 0.0001
Month	45.4	11	< 0.0001
Locality	92.7	19	0.000
Water temp × site type	31.1	6	0.000
pH × site type	12.1	6	0.060
Conductivity × site type	21	6	0.002
Precipitation × site type	18.7	6	0.005

*Key*: Terms: Water temperature × site type, interaction of water temperature and site type; pH × site type, interaction of water temperature and site type; precipitation × site type, interaction of WMO weather station recorded precipitation and site type

### Infection and prevalence

Overall, numbers of snails shedding *Schistosoma* spp. cercariae were very low for *B. forskalii* with a prevalence of *Schistosoma* spp. infection in the snail (proportion of snails that were shedding cercariae) of 0.2% (24/11,989 in total over the study period, overall range by locality 0–6.9%), also low in *B. truncatus* (0.8%, 342/42,500, range by locality 0–6.1%) and relatively high in *Bi. pfeifferi* (3.4%, 79/2290, range for Namari Goungou and Diambala respectively, 1.4–3.7%, Table 2). All *R. natalensis* were negative for *Fasciola* spp. infection. In the glmmTMB of *Bulinus* spp., the total snail abundance showed a significant positive association with the abundance/total number of infected snails shedding cercariae (*χ*^2^ = 30.6, *df* = 1, *P* < 0.001). In testing separately by species, for *B. truncatus* total abundance, the total number of shedding snails was also significant (*χ*^2^ = 33.8, *df* = 1, *P* < 0.001), and similarly for *Bi. pfeifferi* (*χ*^2^ = 19.1, *df* = 1, *P* < 0.001), but not for *B. forskalii*.

### Effect of locality on prevalence of *Schistosoma* spp. infection in snails and on abundance of shedding snails

**Fig. 5:**
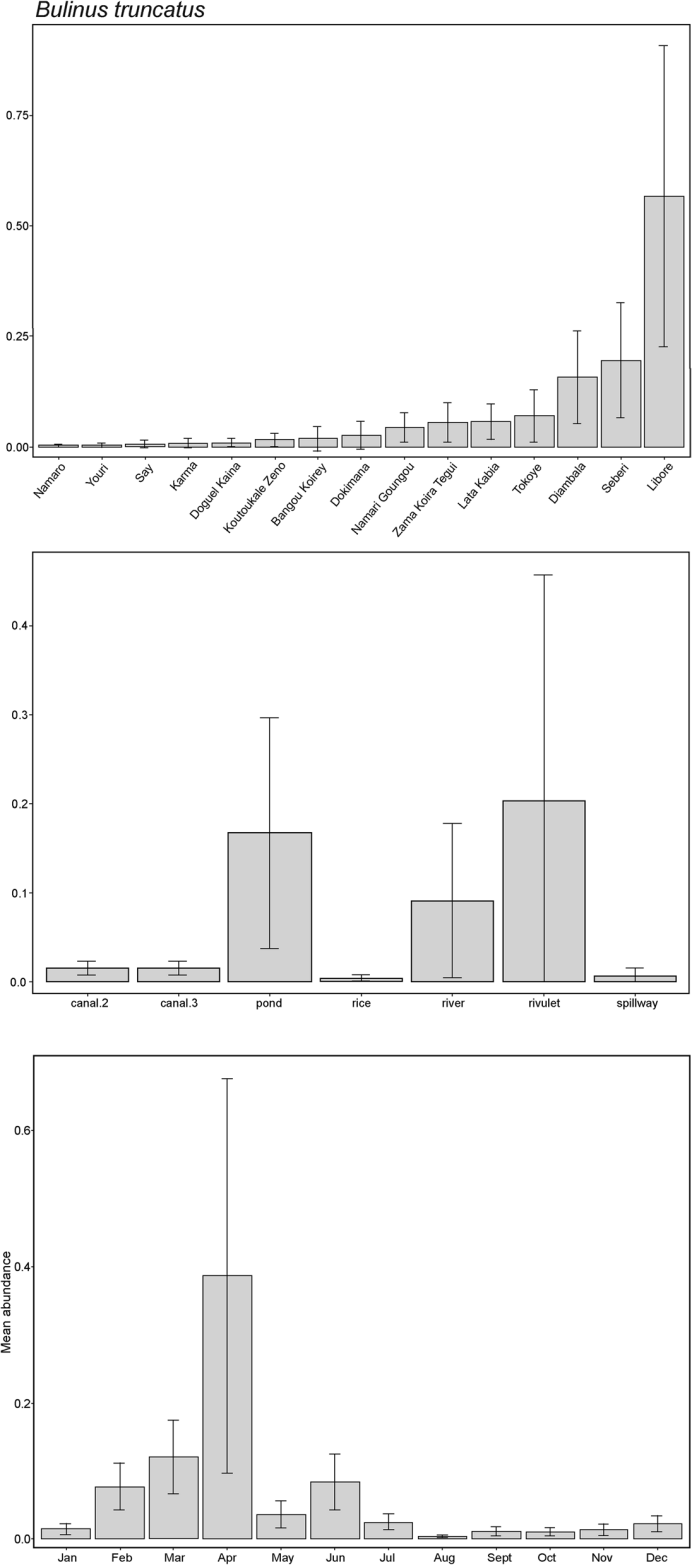
**a** *Bulinus truncatus* modelled abundance of shedding snails by locality (χ2 = 41.7, df = 19, P < 0.002). **b** *B. truncatus* modelled abundance of shedding snails by site type (*χ*^2^ = 15, *df* = 6, *P* < 0.02). **c** *B. truncatus* modelled abundance of shedding snails by month (*χ*^2^ = 62.3, *df* = 11, *P* < 0.001).

Locality was a significant predictor for both proportion of *B. truncatus* that were shedding (*χ*^2^ = 139.4, *df* = 13, *P* < 0.001), and for total abundance of shedding *B. truncatus* (*χ*^2^ = 41.7, *df* = 19, *P* = 0.002), with a positive significant association for Libore only (Fig. 5a, Additional file 1: Table S8). In *B. forskalii*, there was no significant association of locality with either prevalence of *Schistosoma* spp. infection or abundance/total of shedding snails. Some localities without any shedding *B. truncatus* or *B. forskalii* overlapped (Tiaguirie, Tagabati and Yoireize Koira, all with low abundance); similarly some localities with high *Schistosoma* spp. infection prevalence for *B. truncatus* overlapped with high prevalence localities for *B. forskalii* (Tokeye, Libore and Dokimana). For *Bi. pfeifferi*, both *Schistosoma* spp. infection prevalence (*χ*^2^ = 5.1, *df* = 1, *P* = 0.023) and abundance of shedding snails (*χ*^2^ = 8.2, *df* = 1, *P* = 0.004) were significantly higher in Namari Goungou than in Diambala.

### Effect of site type on prevalence of *Schistosoma* spp. infection in snails and on abundance of shedding snails

Site type was a significant predictor for *Schistosoma* spp. infection prevalence in *B. truncatus* (*χ*^2^ = 92.9, *df* = 6, *P* < 0.001). Site type and abundance of shedding *B. truncatus* also showed a significant association (*χ*^2^ = 15, *df* = 6, *P* = 0.02, Fig. 5b), and a significant positive association for ponds (Additional file 1: Table S8). There was no significant association of site type and either *Schistosoma* spp. infection prevalence or abundance of shedding *B. forskalii*. Ponds had the highest proportion of snails shedding *Schistosoma* spp. for both *B. truncatus* and *B. forskalii*, and irrigation canals the lowest for *B. truncatus*, also very low for *B. forskalii* (Table 2). The prevalence of *Schistosoma* spp. infection in *Bi. pfeifferi* was significantly higher in tertiary canals (7%) than in secondary canals (2.5%, *χ*^2^ = 7.1, *df* = 1, *P* = 0.008), but not for total shedding.

### Effect of month on prevalence of *Schistosoma* spp. infection in snails and on abundance of shedding snails

Month was a significant predictor of *Schistosoma* spp. infection prevalence in *B. truncatus* (*χ*^2^ = 18.9, *df* = 11, *P* = 0.06); and of abundance of shedding *B. truncatus* (*χ*^2^ = 62.3, *df* = 11, *P* < 0.001, Fig. 5c), with a significant positive association of shedding *B. truncatus* and months February to April, and June, and a significant negative association for August (Additional file 1: Table S8). Prevalence of *Schistosoma* spp. infection in *B. forskalii* was more variable by month, with no shedding snails collected in April or November in any year. Month was a significant predictor of prevalence of *Schistosoma* spp. infection in *B. forskalii* (*χ*^2^ = 29.4, *df* = 11, *P* = 0.002), but not of abundance of shedding *B. forskalii*. In *Bi. pfeifferi*, the number of infected snails found month by month was also variable. Month was a significant predictor of prevalence of *Schistosoma* spp. infection (*χ*^2^ = 24.3, *df* = 9, *P* = 0.004), but not for abundance of shedding snails. For August, the month with the highest precipitation (Additional file 1: Table S7), prevalence was low for all snails surveyed.

## Discussion

We found significant evidence of seasonality affecting the abundance of freshwater snails in this study, with higher numbers found in the dry season, and reductions in the wet season (Fig. 4c). While this finding was particularly marked for *B. truncatus* and *R. natalensis*, it was evident across all snail species surveyed. This suggests the observation is due to abiotic factors, likely working in concert; for example, snail displacement in wet months as water levels rise and flow increases, and rain creating turbidity, intensifying impact on snails already being washed away. Rain may also affect cumulative impacts through sudden temperature reduction causing thermal shock in snails, reducing egg-laying success, and dampening post-rain recruitment as overall numbers will be reduced [13]. The changing seasonal environment may also affect experimental sampling. Snails may be more difficult to find in turbid water and when dislodged from vegetation; the search-area may increase as water levels rise, and snails may also accumulate in highly localised areas such as eddies which may be missed. Water speed and precipitation both showed a significant negative association with *Bulinus* spp. abundance, and it is well established that *Bulinus* spp. for example prefer low flow environments [13]. Seasonal patterns could also be influenced by density-dependence in the dry season, or where numbers rise to a tipping point, where they start to decline because of factors like food limitation, or potential aestivation [61]. Findings of greater snail abundance in the dry season are consistent with the published data for *Bulinus* spp. in the NRV [62] and in similar environments such as Burkina Faso and Mali [61, 63], but also in very different climates, including Kenya and Lake Victoria [26, 34], although the latter showed differences between ephemeral and permanent sites.

*Schistosoma* spp. infections in *B. truncatus* were also impacted by seasonality (Fig. 4b, Additional file 1: Table S8). Month was a significant predictor of both prevalence of *Schistosoma* spp. infection and overall abundance of shedding *B. truncatus*, with positive associations of shedding snail counts with the dry season and negative with the wet season. Previous work in the NRV has found higher infection in *B. truncatus* in dry season months [62], and here we have statistically confirmed this trend through longitudinal analysis. In a parallel study (Pennance et al. unpublished data), the majority of the shedding snails from this survey were confirmed using molecular markers as infected with the cattle schistosome *S. bovis*, with fewer shedding the human-infecting *S. haematobium* or *S. haematobium* group hybrids. As our study has characterized abundance and distribution of *B. truncatus* that are compatible hosts for *Schistosoma* spp., these data provide a proxy of animal and human schistosomiasis transmission risk. The findings of a higher abundance of *B. truncatus* and higher numbers shedding in the dry season have clear implications for monitoring of transmission for *Schistosoma* spp., and could for example, contribute to an evidence-based snail control programme to tackle interruption of transmission in the region.

We also found a statistically significant correlation between the overall abundance and the total number of infected snails for both *B. truncatus* and *Bi. pfeifferi.* This relationship was not evident for *B. forskalii*, but this may be a sampling effect as infection rates were much lower, consistent with published findings [24], including for the NRV [8, 10]. There is often little evidence of a relationship between snail abundance and the number of infected snails [34, 36]. Here however, monthly sampling has allowed sound resolution of the rates of infection.

Infection in *Bi. pfeifferi*, while variable by month and year, was comparatively high overall, consistent with previous reports in the NRV [21, 44], and in the Niger River catchment in neighbouring Mali [63–65]. It is not clear why this is the case. *Biomphalaria pfeifferi* has a restricted distribution in the NRV, consistent with its recent establishment [46]. It is present downstream of Kandadji Dam, currently under construction. Dam building has previously contributed to the spread of *S. mansoni* as large open water bodies are favourable habitats for *Biomphalaria* spp. [23]; therefore, this dam could also facilitate an increase in *S. mansoni* through the NRV [44]. Moreover *Bi. pfeifferi* could potentially spread further through the NRV regardless of the dam, thereby increasing the risk of *S. mansoni* transmission, and has in fact recently appeared in several more villages (A. Garba, personal communication).

Another key finding is that both prevalence of *Schistosoma* spp. in *B. truncatus* and abundance of shedding *B. truncatus* were significantly higher in ponds (Fig. 5a), suggesting a higher transmission potential in these habitats. Most of the *Bulinus* spp. infections were *S. bovis* (Pennance et al. unpublished data), this may be due to greater water contact by cattle in ponds *versus* other kinds of water contact sites, such as irrigation canals. Wood et al. [66] found a significant correlation of site area and risk of *S. haematobium* infection in Senegal, attributed to more contiguous snail habitat in larger sites. Potentially a similar effect is occurring in the present study in ponds *versus* canals for example. Ponds had the highest average temperatures of 28.5 °C (Additional file 1: Table S7), close to the optimal temperature for schistosome development [41, 67], potentially facilitating successful infection of intermediate host snails. The average temperatures in irrigation canals in contrast is optimal for *Bulinus* spp. (26–27 °C [68]), which may contribute to high abundances of snails in these environments but lower levels of infection. However, snails may also move deeper into a water body with an increase in temperature; therefore, the relationship between snail microhabitat, temperature and infection may be complex. We also found relatively high (although variable) prevalence of *Schistosoma* spp. infection in *B. truncatus* in rivulets, and for *Bi. pfeifferi*, tertiary canals contained proportionally more infected snails, which being dirt-lined are closer to a natural habitat than secondary canals. This presents a picture of higher infection in (more) natural habitats. Variables such as amount of aquatic vegetation may affect the abundance of infected snails, observed as a key factor determining snail presence in other studies [64–66, 69]. This may also occur at the locality level, e.g. Libore is a densely populated area downstream of Niamey, a region previously recorded as hyper-endemic [70] and had significantly highest numbers of infected *B. truncatus* (Fig. 5a). Further Libore was one of several localities with high prevalence across years for both *B. truncatus* and *B. forskalii*. An association of high densities of schistosome-infected snails and high levels of human water contact has previously been observed [64, 70], ascribed to water contamination producing greater growth of aquatic vegetation, favourable for snails. Overall, as shedding *B. truncatus* were found at all site types surveyed, this indicates a range of habitats in the NRV play a role in transmission. Diverse environs and sub-habitats here may result in increased risk of infection of *Schistosoma* spp. in the NRV.

Other spatial trends were evident in the data. High snail abundance in irrigation canals was evident across all species (Table 2). Consistent with this, localities with extensive irrigation, such as Namari Goungou and Seberi, had particularly high snail abundance. Irrigation may represent denser snail habitat, as snails can inhabit both the rice paddies and their adjoining network of canals. Sampling bias may have contributed to the high abundances found in the canals as it may be easier to find snails present in a canal than in a pond, although rivulets, a more similar geography to canals, had low abundances of snails. Differences in site type associations by species were evident; for example, *B. truncatus* was more abundant in secondary than tertiary canals, and rare in rice paddy sites and spillways, whereas *B. forskalii* showed the opposite trends, more abundant in tertiary than secondary canals and common in rice paddies (Fig 5b). This is consistent with knowledge of their general habitat associations, *B. forskalii* being more likely to occur in open habitats such as rice paddies (F. Allan, unpublished observations). Patchiness in the distribution of snails was also evident; snail abundance varied by locality, and some snail species were not present at some localities. Many factors come into play here, snail abundance (and *Schistosoma* spp. prevalence in snails) depend on multiple factors acting in various combinations [71]. Snail modelling has previously looked at snail populations as homogenous [72] but this study, like others shows the need to account for spatial and temporal heterogeneity [61]. Future monitoring of freshwater snails and schistosomes in NRV will need to take into account both seasonality and variable snail distribution [38, 61].

Several limitations to our study and directions for additional work require discussion. A key limitation was that site selection and sampling targeted sites with known high abundances of snails. This was accounted for as far as possible in statistical analysis, but can make comparative analysis problematic, and may have introduced bias. As the survey did not perform density sampling [39, 66], we cannot draw strong conclusions on relative abundances of the different species surveyed. Sampling bias therefore may be substantially affecting relative counts, for example *B. forskalii*, being much smaller than *B. truncatus*, may be missed (as may small juvenile snails and hatchlings of all species). Further, in a parallel study 5 *B. truncatus* from a subset of 137 from our survey were re-identified as *B. globosus* using *cox*1 and ITS molecular markers ([47], Pennance et al. unpublished data). Due to similar morphology of the two species, field identification can be challenging. Therefore, some snails identified as *B. truncatus* may be *B. globosus*, and the latter species may also contribute to transmission of *Schistosoma* spp. in the region. However, numbers of *B. truncatus* overall were much higher than *B. globosus*. Also, *B. truncatus* was substantially more abundant than *B. forskalii*, and the species primarily involved in *Schistosoma* spp. transmission. *B. truncatus* therefore is the main intermediate host in the region. Regarding monitoring of schistosome infections, dissection of snails or use of molecular markers to check for prepatent infections could provide a more accurate and less time-intensive alternative to our method of additional shedding attempts on snails several weeks after collection. Other factors could significantly affect the abundance and distribution of the snail species surveyed that were not measured, such as submerged vegetation [64–66, 69, 73–75]. Some site types might have had more vegetation present, resulting in higher snail abundances, but this would require further analysis with remote sensing data and spatial statistical models [76, 77]. Some localities (Tokeye, Libore, and Dokimana) had high infection prevalence (across multiple years) for both *B. truncatus* and *B. forskalii*, and additional spatial analysis is needed to understand why. In terms of other additional work, hydrological analysis could enhance our understanding of schistosomiasis transmission in the NRV, for example, to model potential accumulation of snails at particular microhabitats or up and down-stream dispersal post-flooding [78–80]. Geomorphology like circumscription of sites in ponds for example combined with localised water flow patterns could influence retention levels for cercariae [38, 66], requiring further investigation. A multidisciplinary approach would see great advances in accurate mapping of schistosomiasis transmission.

## Conclusions

Whether affecting the human population or our livestock, schistosomiasis transmission is dependent on the presence of compatible snail intermediate hosts at water-contact sites. An intensive, longitudinal approach to snail sampling has provided this study with the resolution to reveal significant seasonal and spatial variation in snail infection and abundance, which could be used in an evidence-based intervention strategy to control schistosomiasis in the Niger River Valley. The impact of season on *B. truncatus* is a key finding with these snails acting as the main intermediate host for species of the *Schistosoma haematobium* group in the region. Within the dry season *B. truncatus* was more abundant and showed higher levels of schistosome infections. These results could inform timing of praziquantel administration among the human population and support any other behavioral or snail control interventions. As monitoring of snails is often overlooked, these data show how crucial local-scale snail surveys are to fully understanding transmission dynamics and mapping schistosomiasis risk in a given region and contribute to any future attempts at adjunct interventions through control of snail populations.

## Supplementary information

**Additional file 1: Table S1.** Snail survey sites (coordinates in decimal, WGS84). **Figure S1.** Predicted counts by site type, *B. truncatus.* **Figure S2.** Estimated slope of *Bulinus* spp. with temperature by site type. Here a 1 degree increase in temperature equals decrease in abundance in rivulet and river, rice paddies, ponds and irrigation canals no real change although in latter—v slight increase in abundance with temp, spillway and stream (I site)—increase in abundance with temperature. **Figure S3.** Estimated slope of *Bulinus* spp. with water speed. **Table S2.** *B. truncatus* glmmTMB negative binomial model summary output. **Table S3.** *B. forskalii* glmmTMB negative binomial model summary output. **Table S4.** *Radix natalensis* glmmTMB negative binomial model summary output. **Table S5.** *Biomphalaria pfeifferi* glmmTMB negative binomial model summary output. **Table S6.** *Bulinus* spp glmmTMB negative binomial model summary output. **Table S7.** Water chemistry and physical variable data, including USDA (WMO) weather station data, A: averaged by month, and B: by site type (final year of ground collected data missing due to equipment failure). **Table S8.** Shedding/infected *B. truncatu*s glmmTMB negative binomial model summary output.

## Data Availability

The complete dataset, metadata and scripts for data analysis are available at GitHub (https://github.com/howlerMoonkey/Niger_snail_survey_data_analysis). Collection data for voucher snail specimens and schistosome cercariae are available at the NHM data portal (http://data.nhm.ac.uk) and the SCAN website (http://scan.myspecies.info).

## Abbreviations

*P*: *P*-value
*df*: degrees of freedom
*χ*^2^: Chi-square value
*n*: total count
BT: *Bulinus truncatus*
BF: *Bulinus forskalii*
BP: *Biomphalaria pfeifferi*
RN: *Radix natalensis*
NRV: Niger River Valley
Canal 2: secondary irrigation canal
Canal 3: tertiary irrigation canal
Rice: rice paddy site
WMO: USDA Foreign Agricultural Service weather meteorological office

## References

[1] Vos T, Abajobir AA, Abate KH, Abbafati C, Abbas KM, Murray CJL (2017). Global, regional, and national incidence, prevalence, and years lived with disability for 328 diseases and injuries for 195 countries, 1990–2016: a systematic analysis for the Global Burden of Disease Study. Lancet.

[2] Sturrock RF, Jordan P, Webbe G, Sturrock RF (1993). The intermediate hosts and host-parasite relationships. Human schistosomiasis.

[3] Bustinduy A, King C, Scott J, Appleton S, Sousa-Figueiredo J, Betson M, Stothard J (2014). HIV and schistosomiasis co-infection in African children. Lancet Infect Dis..

[4] Christinet V, Lazdins-Helds J, Stothard J, Reinhard-Rupp J (2016). Female genital schistosomiasis (FGS): from case reports to a call for concerted action against this neglected gynaecological disease. Int J Parasitol..

[5] Hotez PJ, Harrison W, Fenwick A, Bustinduy AL, Ducker C, Sabina Mbabazi P (2019). Female genital schistosomiasis and HIV/AIDS: reversing the neglect of girls and women. PLoS Negl Trop Dis..

[6] Kayuni S, Lampiao F, Makaula P, Juziwelo L, Lacourse EJ, Reinhard-Rupp J (2019). A systematic review with epidemiological update of male genital schistosomiasis (MGS): a call for integrated case management across the health system in sub-Saharan Africa. Parasite Epidemiol Control.

[7] Standley CJ, Dobson AP, Stothard JR, Rokni MB (2012). Out of animals and back again: schistosomiasis as a zoonosis in Africa. schistosomiasis.

[8] Labbo R, Djibrilla A, Zamankaa H, Garba A, Chippaux J-PB (2007). *forskalii*: a new potential intermediate host for *Schistosoma haematobium* in Niger. Trans R Soc Trop Med Hyg..

[9] Labbo R, Ernould JC, Djibrila A, Bangre M, Labbo R. Epidémiologie de la schistosomose à *Schistosoma bovis*: détermination des charges parasitaires, de la période de transmission et des sites de contamination a partir des enquêtes d’abattoir—document CERMES No. 01/01 Niamey, Niger; 2001. p. 13.

[10] Bremond P, Vera C, Labbo R, Sellin E, Jourdane J, Sellin B. Variability in the compatibility between *Schistosoma haematobium*, *S. bovis*, *S. curassoni* and bulinid snails in Niger: implications on interactions between parasites. In: VIIIth international congress of parasitology (ICOPA VIII), 10–14 October 1994, Izmir, Turkey (Abstracts, vol. 1); 1994. p. 207.

[11] Bremond P, Sellin B, Sellin E, Nameoua B, Labbo R (1993). Evidence for the introgression of the human parasite *Schistosoma haematobium* by genes from *Schistosoma bovis*, Niger. C R Acad Sci III.

[12] Moné H, Mouahid G, Morand S (1999). The distribution of *Schistosoma bovis* Sonsino, 1876 in relation to intermediate host mollusc-parasite relationships. Adv Parasitol.

[13] Rollinson D, Stothard JR, Southgate VR (2001). Interactions between intermediate snail hosts of the genus *Bulinus* and schistosomes of the *Schistosoma haematobium* group. Parasitology.

[14] Southgate VR, Knowles RJ (1975). Observations on *Schistosoma bovis* Sonsino, 1976. J Nat Hist.

[15] Southgate VR, Knowles RJ (1975). The intermediate hosts of *Schistosoma bovis* in western Kenya. Trans R Soc Trop Med Hyg..

[16] Tian-Bi Y-N, Webster B, Konan CK, Allan F, Diakité NR, Ouattara M (2019). Molecular characterization and distribution of *Schistosoma* cercariae collected from naturally infected bulinid snails in northern and central Côte d’Ivoire. Parasites Vectors.

[17] Leger E, Garba A, Hamidou AA, Webster BL, Pennance T, Rollinson D, Webster JP (2016). Introgressed animal schistosomes *Schistosoma curassoni* and *S. bovis* naturally infecting humans. Emerg Infect Dis.

[18] Webster BL, Diaw OT, Seye M, Webster JP, Rollinson D (2013). Introgressive hybridization of *Schistosoma haematobium* group species in Senegal: species barrier break down between ruminant and human schistosomes. PLoS Negl Trop Dis.

[19] Huyse T, Webster B, Geldof S, Stothard J, Diaw O, Polman K, Rollinson D (2009). Bidirectional introgressive hybridization between a cattle and human schistosome species. PloS Pathog.

[20] Rollinson D, Southgate VR, Vercruysse J, Moore PJ (1990). Observations on natural and experimental interactions between *Schistosoma bovis* and *S. curassoni* from West Africa. Acta Trop..

[21] Garba A, Barkiré N, Djibo A, Lamine MS, Sofo B, Gouvras AN (2010). Schistosomiasis in infants and preschool-aged children: infection in a single *Schistosoma haematobium* and a mixed *S. haematobium*–*S. mansoni* foci of Niger. Acta Trop.

[22] Chitsulo LD, Engels A, Montresor A, Savioli L (2000). The global status of schistosomiasis and its control. Acta Trop..

[23] Steinmann P, Keiser J, Bos R, Tanner M, Utzinger J (2006). Schistosomiasis and water resources development: systematic review, meta-analysis, and estimates of people at risk. Lancet Infect Dis.

[24] Brown DS (1994). Freshwater snails of Africa and their medical importance.

[25] Comité Permanent Inter-états de Lutte contre la Sécheresse dans le Sahel (CILSS). Landscapes of West Africa—a window on a changing world. Ouagadougou, Burkina Faso: CILSS; 2016. p. 219. 10.5066/f7n014qz.

[26] Gouvras AN, Allan F, Kinung’hi S, Rabone M, Emery A, Angelo T (2017). Longitudinal survey on the distribution of *Biomphalaria sudanica* and *B. choanomophala* in Mwanza region, on the shores of Lake Victoria, Tanzania: implications for schistosomiasis transmission and control. Parasites Vectors.

[27] Allan F, Sousa-Figueiredo JC, Emery A, Rossely P, Mirante C, Rollinson D (2017). Mapping freshwater snails in north-western Angola: distribution, identity and molecular diversity of medically important taxa. Parasites Vectors..

[28] Senghor B, Diaw OT, Doucoure S, Seye M, Talla I, Diallo A, Tidiane Bâ B, Sokhna C (2015). Study of the snail intermediate hosts of urogenital schistosomiasis in Niakhar, region of Fatick, West central Senegal. Parasites Vectors..

[29] Labbo R, Boulanger D, Bremond P, Chippaux J-P (2007). Infestation expérimentale de caprins par *Schistosoma bovis* et *S. curassoni*: effets pathogènes comparés. Parasite.

[30] Mutuku MW, Laidemitt MR, Beechler BR, Mwangi IN, Otiato FO, Agola EL (2019). A search for snail-related answers to explain differences in response of *Schistosoma mansoni* to praziquantel treatment among responding and persistent hotspot villages along the Kenyan shore of Lake Victoria. Am J Trop Med Hyg.

[31] Stensgaard AS, Jørgensen A, Kabatereine NB, Rahbek C, Kristensen TK (2006). Modeling freshwater snail habitat suitability and areas of potential snail-borne disease transmission in Uganda. Geospat Health.

[32] Moser W, Greter H, Schindler C, Allan F, Ngandolo BNR, Moto DD, Utzinger J, Zinsstag J (2014). The spatial and seasonal distribution of *B. truncatus*, *B. forskalii* and *Bi. pfeifferi*, the intermediate host snails of schistosomiasis, in N’Djamena, Chad. Geospat Health.

[33] Diakité NR, N’Zi KG, Ouattara M, Coulibaly JT, Saric J, Yao PK, Hattendorf J, Utzinger J, N’Goran EK (2018). Association of riverine prawns and intermediate host snails and correlation with human schistosomiasis in two river systems in south-eastern Côte d’Ivoire. Parasitology.

[34] Muhoho ND, Katsumata T, Kimura E, Migwi DK, Mutua WR, Kiliku FM, Habe S, Aoki Y (1997). Cercarial density in the river of an endemic area of *Schistosomiasis haematobium* in Kenya. Am J Trop Med Hyg..

[35] Gryseels B (1991). The epidemiology of schistosomiasis in Burundi and its consequences for control. Trans R Soc Trop Med Hyg..

[36] Klumpp, R. A study of the transmission of *Schistosoma haematobium* in Volta Lake, Ghana. Ph.D. thesis, London School of Hygiene & Tropical Medicine; 1983. 10.17037/pubs.00682402.

[37] Woolhouse ME, Chandiwana SK (1989). Spatial and temporal heterogeneity in the population dynamics of *Bulinus globosus and Biomphalaria pfeifferi* and in the epidemiology of their infection with schistosomes. Parasitology.

[38] Ramalli L, Mulero S, Noël H, Chiappini JD, Vincent J (2018). Persistence of schistosomal transmission linked to the Cavu river in southern Corsica since 2013. Eurosurveillance.

[39] Sokolow S, Wood C, Jones I, Lafferty K, Kuris A, Hsieh M, De Leo G (2017). To reduce the global burden of human schistosomiasis, use “old fashioned” snail control. Trends Parasitol..

[40] King CH, Sutherland LJ, Bertsch D (2015). Systematic review and meta-analysis of the impact of chemical-based mollusciciding for control of *Schistosoma mansoni* and *S. haematobium* transmission. PLoS Negl Trop Dis..

[41] Stensgaard AS, Vounatsou P, Sengupta M, Utzinger J (2019). Schistosomes, snails and climate change: current trends and future expectations. Acta Trop..

[42] Gurarie D, King CH, Yoon N, Alsallaq R, Wang X (2016). Seasonal dynamics of snail populations in coastal Kenya: model calibration and snail control. Adv Water Resour..

[43] Ezeamama A, He CL, Shen Y, Yin X-P, Binder SC, Campbell C (2016). Gaining and sustaining schistosomiasis control: study protocol and baseline data prior to different treatment strategies in five African countries. BMC Infect Dis..

[44] Garba A, Labbo R, Tohon Z, Sidiki A, Djibrilla A (2004). Emergence of *Schistosoma mansoni* in the Niger River valley, Niger. Trans R Soc Trop Med Hyg..

[45] Utzinger J, Brattig NW, Kristensen TK (2013). Schistosomiasis research in Africa: how the CONTRAST alliance made it happen. Acta Trop..

[46] Labbo R, Garba A, Louboutin-Croc JP, Ernould JC, Sellin B, Chippaux J-P, Stothard JR (2003). The spread of *Biomphalaria pfeifferi* in the Niger River valley, Niger. Ann Trop Med Parasitol..

[47] Kane RA, Stothard JR, Emery A, Rollinson D (2008). Molecular characterization of freshwater snails in the genus *Bulinus*: a role for barcodes?. Parasites Vectors..

[48] Frandsen F, Christensen NO (1984). An introductory guide to the identification of cercariae from African freshwater snails with special reference to cercariae of trematode species of medical and veterinary importance. Acta Trop..

[49] Gower CM, Shrivastava J, Lamberton PHL, Rollinson D, Webster BL, Emery A (2007). Development and application of an ethically and epidemiologically advantageous assay for the multi-locus microsatellite analysis of *Schistosoma mansoni*. Parasitology..

[50] Emery A, Allan F, Rabone M, Rollinson D (2012). Schistosomiasis collection at NHM (SCAN). Parasites Vectors.

[51] R Core Team. R: a language and environment for statistical computing. In: R version 3.5.3 (2019-03-11) “Great Truth” edn. Vienna: R Foundation for Statistical Computing; 2019. https://www.R-project.org/.

[52] Team R. RStudio: integrated development for R. In: 0.99.903 edn. Boston: RStudio, Inc.; 2015.

[53] Zuur A, Ieno EN, Elphick CS (2010). A protocol for data exploration to avoid common statistical problems. Methods Ecol Evol.

[54] Brooks M, Kristensen K, van Benthem K, Magnusson A, Berg C, Nielsen A (2017). glmmTMB balances speed and flexibility among packages for zero-inflated generalized linear mixed modeling. R J.

[55] Blasco-Moreno A, Pérez-Casany M, Puig P, Morante M, Castells E (2019). What does a zero mean? Understanding false, random and structural zeros in ecology. Methods Ecol Evol.

[56] Hartig F. DHARMa: residual diagnostics for hierarchical (multi-level/mixed) regression models. R package, version 0.2.4. 2019. http://florianhartig.github.io/DHARMa/.

[57] Burnham KP, Anderson DR, Huyvaert KP (2011). AIC model selection and multimodel inference in behavioral ecology: some background, observations, and comparisons. Behav Ecol Sociobiol..

[58] Mundry R (2011). Issues in information theory-based statistical inference—a commentary from a frequentist’s perspective. Behav Ecol Sociobiol..

[59] Fox J, Weisberg S. Car, R package; 2011. https://cran.r-project.org/web/packages/car/index.html.

[60] Lenth R, Singmann H, Love J, Beurkner P, Herve M. emmeans 2019-04-21; 2019. https://CRAN.R-project.org/package=emmeans.

[61] Perez-Saez J, Mande T, Ceperley N, Bertuzzo E, Mari L, Gatto M, Rinaldo A (2016). Hydrology and density feedbacks control the ecology of intermediate hosts of schistosomiasis across habitats in seasonal climates. Proc Natl Acad Sci USA.

[62] Labbo R, Ernould JC, Djibrilla A, Garba A, Chippaux J-P (2008). Focalisation de la transmission de *Schistosoma haematobium* au sein des périmètres irrigués de la vallée du Niger: importance des facteurs malacologiques. Rev Epidemiol Sante Publique.

[63] Dabo A, Diarra AZ, Machault V, Touré O, Niambélé DS, Kanté A (2015). Urban schistosomiasis and associated determinant factors among school children in Bamako, Mali, West Africa. Infect Dis Poverty.

[64] Madsen H, Coulibaly G, Furu P (1987). Distribution of freshwater snails in the river Niger basin in Mali with special reference to the intermediate hosts of schistosomes. Hydrobiologia..

[65] Madsen H (1992). Ecological studies on the intermediate host snails and the relevance to schistosomiasis control. Mem Inst Oswaldo Cruz.

[66] Wood CL, Sokolow S, Jones I, Chamberlin A, Lafferty KD, Kuris AM, et al. Precision mapping of snail habitat provides a powerful indicator of human schistosomiasis transmission. Proc Natl Acad Sci USA; 2019 (**in press**). 10.1073/pnas.1903698116 PMC6859407 31659025

[67] Appleton CC (1978). Review of literature on abiotic factors influencing the distribution and life cycles of bilharzia intermediate host snails. Malacol Rev..

[68] Shiff CJ (1964). Studies on *Bulinus* (*Phyopsis*) in Rhodesia. The influence of temperature on the intrinsic rate of natural increase. Ann Trop Med Parasitol.

[69] Klumpp R, Chu K (1977). Ecological studies of *Bulinus rohlfsi*, the intermediate host of *Schistosoma haematobium* in the Volta Lake. Bull World Health Organ.

[70] Ernould JC, Kaman AK, Labbo R, Couret D, Chippaux J-P (2000). Recent urban growth and urinary schistosomiasis in Niamey, Niger. Trop Med Int Health.

[71] Utzinger J, Tanner M (2000). Microhabitat preferences of *Biomphalaria pfeifferi* and *R. natalensis* in a natural and a man-made habitat in southeastern Tanzania. Mem Inst Oswaldo Cruz.

[72] Gurarie D, Lo N, Ndeffo-Mbah M, Durham D, King C (2018). The human-snail transmission environment shapes long term schistosomiasis control outcomes: implications for improving the accuracy of predictive modeling. PLoS Negl Trop Dis..

[73] Coulibaly G, Diallo M, Madsen H, Dabo A, Traoré M, Keita S (2004). Comparison of schistosome transmission in a single- and a double-cropped area in the rice irrigation scheme, ‘Office du Niger’, Mali. Acta Trop.

[74] Scott D, Senker K, England E (1982). Epidemiology of human *Schistosoma haematobium* infection around Volta Lake, Ghana, 1973–75. Bull World Health Organ.

[75] Thomas J, Tait A (1984). Control of the snail hosts of schistosomiasis by environmental manipulation: a field and laboratory appraisal in the Ibadan Area, Nigeria. Philos Trans R Soc Lond B Biol Sci.

[76] Kulinkina AV, Walz Y, Koch M, Biritwum N-K, Utzinger J, Naumova EN (2018). Improving spatial prediction of *Schistosoma haematobium* prevalence in southern Ghana through new remote sensors and local water access profiles. PLoS Negl Trop Dis..

[77] Walz Y, Wegmann M, Dech S, Vounatsou P, Poda J-N, N’Goran EK, Utzinger J, Raso G (2015). Modeling and validation of environmental suitability for schistosomiasis transmission using remote sensing. PLoS Negl Trop Dis.

[78] Perez-Saez J, Mande T, Larsen J, Ceperley N, Rinaldo A (2017). Classification and prediction of river network ephemerality and its relevance for waterborne disease epidemiology. Adv Water Resour.

[79] Ciddio M, Mari L, Gatto M, Rinaldo A, Casagrandi R (2015). The temporal patterns of disease severity and prevalence in schistosomiasis. Chaos.

[80] Clennon JA, King CH, Muchiri EM, Kitron U (2007). Hydrological modelling of snail dispersal patterns in Msambweni, Kenya and potential resurgence of *Schistosoma haematobium* transmission. Parasitology.

