# Supplementary material for: Freshwater snails of biomedical importance in the Niger River Valley: evidence of temporal and spatial patterns in abundance, distribution and infection with Schistosoma spp
Source: Parasit Vectors. 2019 Oct 22;12:498. doi: 10.1186/s13071-019-3745-8 (PMC6805334; doi:10.1186/s13071-019-3745-8)
Supplement: Supplementary file 1 — Additional file 1: Table S1. Snail survey sites (coordinates in decimal, WGS84). Figure S1. Predicted counts by site type, B. truncatus. Figure S2. Estimated slope of Bulinus spp. with temperature by site type. Here a 1 degree increase in temperature equals decrease in abundance in rivulet and river, rice paddies, ponds and irrigation canals no real change although in latter—v slight increase in abundance with temp, spillway and stream (I site)—increase in abundance with temperature. Figure S3. Estimated slope of Bulinus spp. with water speed. Table S2. B. truncatus glmmTMB negative binomial model summary output. Table S3. B. forskalii glmmTMB negative binomial model summary output. Table S4. Radix natalensis glmmTMB negative binomial model summary output. Table S5. Biomphalaria pfeifferi glmmTMB negative binomial model summary output. Table S6. Bulinus spp glmmTMB negative binomial model summary output. Table S7. Water chemistry and physical variable data, including USDA (WMO) weather station data, A: averaged by month, and B: by site type (final year of ground collected data missing due to equipment failure). Table S8. Shedding/infected B. truncatus glmmTMB negative binomial model summary output. [file 13071_2019_3745_MOESM1_ESM.docx]

**Additional file 1: Table S1. Snail survey sites** (coordinates in decimal, WGS84)

| **site_code** | **district** | **locality** | **site_name** | **site_type** | **latitude** | **longitude** |
| --- | --- | --- | --- | --- | --- | --- |
| 382865 | Kollo | Bangou Koirey | bangou koirey 1 bras du fleuve | rivulet | 13.60331 | 1.90767 |
| 382864 | Kollo | Bangou Koirey | bangou koirey 2 bras du fleuve | rivulet | 13.60475 | 1.90509 |
| 1658554 | Kollo | Bangou Koirey | bangou koirey 3 bras du fleuve | rivulet | 13.60993 | 1.90371 |
| 382866 | Tillaberi | Diambala | diambala 1 canal tertiaire | canal.3 | 14.31071 | 1.29644 |
| 382867 | Tillaberi | Diambala | diambala 2 canal tertiaire | canal.3 | 14.31055 | 1.28587 |
| 382868 | Tillaberi | Diambala | diambala 3 canal secondaire | canal.2 | 14.30949 | 1.28911 |
| 382869 | Tillaberi | Diambala | diambala 4 canal secondaire | canal.2 | 14.30968 | 1.29359 |
| 382870 | Tillaberi | Diambala | diambala 5 canal tertiaire | canal.3 | 14.30989 | 1.29365 |
| 382871 | Tillaberi | Diambala | diambala 6 canal secondaire | canal.2 | 14.31036 | 1.29175 |
| 382872 | Tillaberi | Diambala | diambala 7 canal tertiaire | canal.3 | 14.31047 | 1.29181 |
| 382874 | Say | Doguel Kaina | doguel kaina 1 bras du fleuve | rivulet | 13.28931 | 2.32526 |
| 382875 | Say | Doguel Kaina | doguel kaina 2 canal tertiaire | canal.3 | 13.28724 | 2.32726 |
| 382876 | Say | Doguel Kaina | doguel kaina 3 riziere | rice.p | 13.28718 | 2.32726 |
| 436558 | Say | Doguel Kaina | doguel kaina 4 fleuve | river | 13.29273 | 2.32335 |
| 601671 | Say | Doguel Kaina | doguel kaina 5 canal secondaire | canal.2 | 13.28259 | 2.33279 |
| 382877 | Say | Dokimana | dokimana 1 mare nordest | pond | 13.03523 | 2.3432 |
| 382878 | Say | Dokimana | dokimana 2 mare sudouest | pond | 13.03144 | 2.34215 |
| 382879 | Say | Gantchi Bassarou | gantchi bassarou 1 mare | pond | 13.17606 | 2.35497 |
| 382880 | Say | Gantchi Bassarou | gantchi bassarou 2 riziere | rice.p | 13.17232 | 2.34953 |
| 382881 | Kollo | Karma | karma 1 fleuve | river | 13.66431 | 1.82411 |
| 382882 | Kollo | Karma | karma 2 canal secondaire | canal.2 | 13.66373 | 1.824866 |
| 7920104 | Kollo | Karma | karma 3 canal tertiaire | canal.3 | 13.66367 | 1.82437 |
| 382884 | Kollo | Karma | karma 4 riziere | rice.p | 13.66344 | 1.82505 |
| 551014 | Kollo | Karma | karma 5 canal tertiaire | canal.3 | 13.66395 | 1.82468 |
| 487390 | Kollo | Karma | karma 6 bras du fleuve | rivulet | 13.66605 | 1.80992 |
| 382885 | Say | Kohan | kohan garantche 1 fleuve | river | 13.31996 | 2.29661 |
| 382887 | Say | Koutoukale | koutoukale zeno 1 canal secondaire | canal.2 | 13.68637 | 1.7452 |
| 382883 | Say | Koutoukale | koutoukale zeno 2 canal tertiaire | canal.3 | 13.6862 | 1.74518 |
| 382889 | Say | Koutoukale | koutoukale zeno 3 mare | pond | 13.6916 | 1.74321 |
| 487391 | Say | Koutoukale | koutoukale zeno 4 canal secondaire | canal.2 | 13.68351 | 1.75121 |
| 601670 | Say | Koutoukale | koutoukale zeno 5 canal tertiaire | canal.3 | 13.68381 | 1.75045 |
| 382891 | Kollo | Lata | lata kabia 1 canal secondaire | canal.2 | 13.74196 | 1.68257 |
| 436559 | Kollo | Lata | lata kabia 2 canal tertiaire | canal.3 | 13.74193 | 1.68262 |
| 382893 | Kollo | Lata | lata kabia 3 riziere | rice.p | 13.74199 | 1.68258 |
| 382892 | Kollo | Lata | lata kabia 4 mare | pond | 13.7425 | 1.68173 |
| 436560 | Kollo | Lata | lata kabia 5 canal tertiaire | canal.3 | 13.7561 | 1.67038 |
| 436561 | Kollo | Lata | lata kabia 6 deversoir | spillway | 13.75618 | 1.67031 |
| 487394 | Kollo | Lata | lata kabia 7 canal secondaire | canal.2 | 13.76104 | 1.67135 |
| 487395 | Kollo | Lata | lata kabia 8 canal secondaire | canal.2 | 13.76385 | 1.66861 |
| 382895 | Kollo | Libore | libore 1 canal secondaire | canal.2 | 13.40273 | 2.18918 |
| 382896 | Kollo | Libore | libore 2 canal secondaire | canal.2 | 13.40304 | 2.18897 |
| 382897 | Kollo | Libore | libore 3 canal tertiaire | canal.3 | 13.4034 | 2.18862 |
| 382898 | Kollo | Libore | libore 4 riziere | rice.p | 13.40342 | 2.18843 |
| 360829 | Kollo | Libore | libore 5 canal tertiaire | canal.3 | 13.39008 | 2.19711 |
| 436562 | Kollo | Libore | libore 6 canal secondaire | canal.2 | 13.39002 | 2.19714 |
| 360779 | Kollo | Libore | libore 6 riziere | rice.p | 13.39001 | 2.19713 |
| 382899 | Kollo | Libore | libore 7 canal secondaire | canal.2 | 13.39452 | 2.19297 |
| 382900 | Kollo | Libore | libore 8 canal secondaire | canal.2 | 13.40417 | 2.18802 |
| 382901 | Kollo | Libore | libore 9 canal secondaire | canal.2 | 13.38805 | 2.19913 |
| 382902 | Tillaberi | Namari Goungou | namari goungou 1 canal tertiaire | canal.3 | 14.34843 | 1.24819 |
| 382903 | Tillaberi | Namari Goungou | namari goungou 2 canal tertiaire | canal.3 | 14.34942 | 1.24808 |
| 382904 | Tillaberi | Namari Goungou | namari goungou 3 canal secondaire | canal.2 | 14.34818 | 1.25013 |
| 382905 | Tillaberi | Namari Goungou | namari goungou 4 canal tertiaire | canal.3 | 14.34492 | 1.25329 |
| 382906 | Tillaberi | Namari Goungou | namari goungou 5 canal tertiaire | canal.3 | 14.34615 | 1.24468 |
| 487296 | Tillaberi | Namari Goungou | namari goungou 6 canal secondaire | canal.2 | 14.34488 | 1.24571 |
| 382907 | Kollo | Namaro | namaro 1 canal secondaire | canal.2 | 13.71095 | 1.69746 |
| 382908 | Kollo | Namaro | namaro 2 canal tertiaire | canal.3 | 13.71124 | 1.69775 |
| 382909 | Kollo | Namaro | namaro 3 mare ouest | pond | 13.70943 | 1.69807 |
| 487396 | Kollo | Namaro | namaro 4 fleuve | river | 13.68974 | 1.71444 |
| 487397 | Kollo | Namaro | namaro 5 mare est | pond | 13.7102 | 1.69744 |
| 382911 | Say | Say | say 2 deversoir | spillway | 13.10468 | 2.35263 |
| 382912 | Say | Say | say 3 canal secondaire | canal.2 | 13.09918 | 2.35073 |
| 487398 | Say | Say | say 4 fleuve | river | 13.09848 | 2.37032 |
| 382913 | Kollo | Seberi | seberi 1 canal secondaire | canal.2 | 13.29359 | 2.34353 |
| 382914 | Kollo | Seberi | seberi 2 canal secondaire | canal.2 | 13.29434 | 2.34215 |
| 382915 | Kollo | Seberi | seberi 3 canal tertiaire | canal.3 | 13.29699 | 2.33337 |
| 382916 | Kollo | Seberi | seberi 4 canal tertiaire | canal.3 | 13.3038 | 2.33241 |
| 382917 | Kollo | Seberi | seberi 5 canal secondaire | canal.2 | 13.31099 | 2.32554 |
| 382918 | Kollo | Seberi | seberi 6 canal secondaire | canal.2 | 13.31313 | 2.32354 |
| 382920 | Kollo | Tagabati | tagabati 1 fleuve | river | 13.63102 | 1.87492 |
| 382921 | Kollo | Tagabati | tagabati 2 bras du fleuve | rivulet | 13.63109 | 1.87452 |
| 487399 | Kollo | Tagabati | tagabati 3 fleuve | river | 13.62944 | 1.87799 |
| 382923 | Kollo | Tiaguirire | tiaguirire 1 canal secondaire | canal.2 | 13.27167 | 2.33836 |
| 382922 | Kollo | Tiaguirire | tiaguirire 2 canal tertiaire | canal.3 | 13.27166 | 2.33829 |
| 487400 | Kollo | Tiaguirire | tiaguirire 3 riziere | rice.p | 13.27176 | 2.33823 |
| 382926 | Kollo | Tokeye | tokoye 1 mare | pond | 13.19668 | 2.35522 |
| 382927 | Kollo | Tokeye | tokoye 2 canal secondaire | canal.2 | 13.19224 | 2.36141 |
| 382928 | Kollo | Tokeye | tokoye 3 canal tertiaire | canal.3 | 13.19123 | 2.36171 |
| 487415 | Kollo | Tokeye | tokoye 4 canal secondaire | canal.2 | 13.1925 | 2.36142 |
| 382929 | Kollo | Yoreize Koira | yoreize koira 1 canal secondaire | canal.2 | 13.61844 | 1.87246 |
| 382930 | Kollo | Yoreize Koira | yoreize koira 2 canal tertiaire | canal.3 | 13.61853 | 1.87262 |
| 382931 | Kollo | Yoreize Koira | yoreize koira 3 riziere | rice.p | 13.61857 | 1.87245 |
| 487401 | Kollo | Yoreize Koira | yoreize koira 4 canal tertiaire | canal.3 | 13.61638 | 1.87466 |
| 382932 | Kollo | Youri | youri 1 bras du fleuve | rivulet | 13.33866 | 2.24118 |
| 382933 | Kollo | Youri | youri 2 riziere | rice.p | 13.33507 | 2.24781 |
| 382934 | Kollo | Youri | youri 3 fleuve | river | 13.33371 | 2.24556 |
| 382935 | Kollo | Zama Koira Tegui | zama koira tegui 1 canal secondaire | canal.2 | 13.70169 | 1.71942 |
| 382936 | Kollo | Zama Koira Tegui | zama koira tegui 2 canal tertiaire | canal.3 | 13.70186 | 1.71945 |
| 382937 | Kollo | Zama Koira Tegui | zama koira tegui 3 riziere | rice.p | 13.70183 | 1.71938 |
| 436563 | Kollo | Zama Koira Tegui | zama koira tegui 4 canal secondaire | canal.2 | 13.70432 | 1.72169 |
| 487402 | Kollo | Zama Koira Tegui | zama koira tegui 5 deversoir | spillway | 13.70023 | 1.71885 |
| 487403 | Kollo | Zama Koira Tegui | zama koira tegui 6 canal tertiaire | canal.3 | 13.709 | 1.71364 |


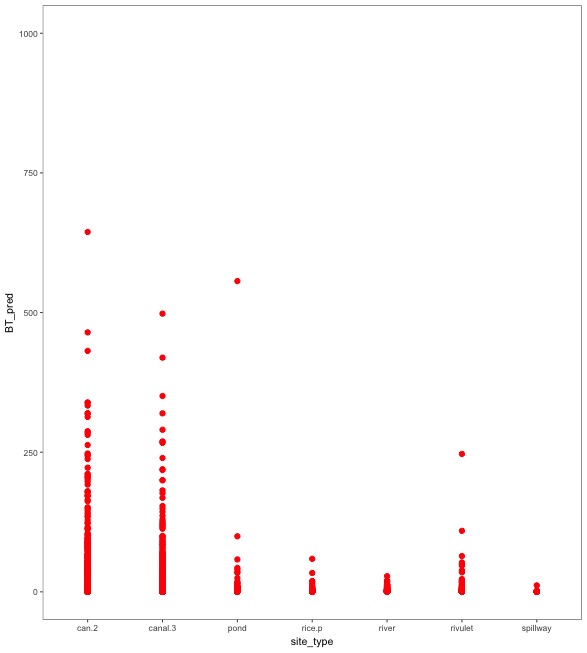


**Additional file 1: Figure S1**: Predicted counts by site type, *B. truncatus*


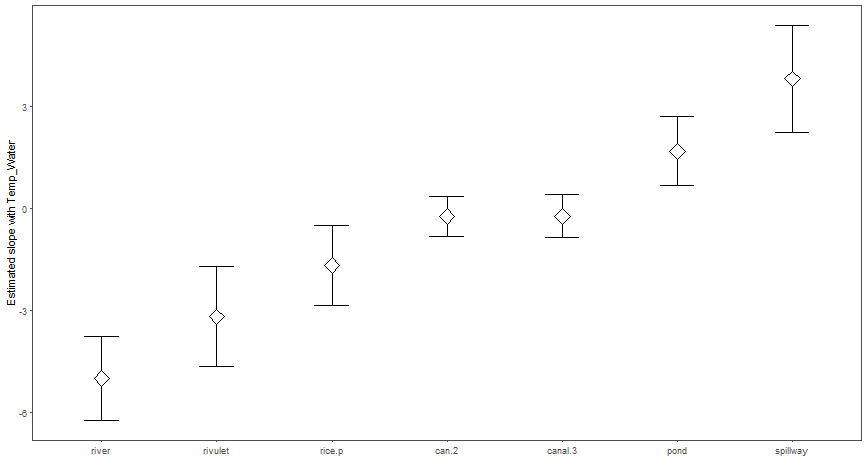


**Additional file 1: Figure S2.** Estimated slope of Bulinus spp with temperature by site type. 1 degree increase in temperature equals decrease in abundance in rivulet and river, rice paddies, ponds and irrigation canals no real change although in latter- v slight increase in abundance with temp, spillway and stream (I site)- increase in abundance with temperature


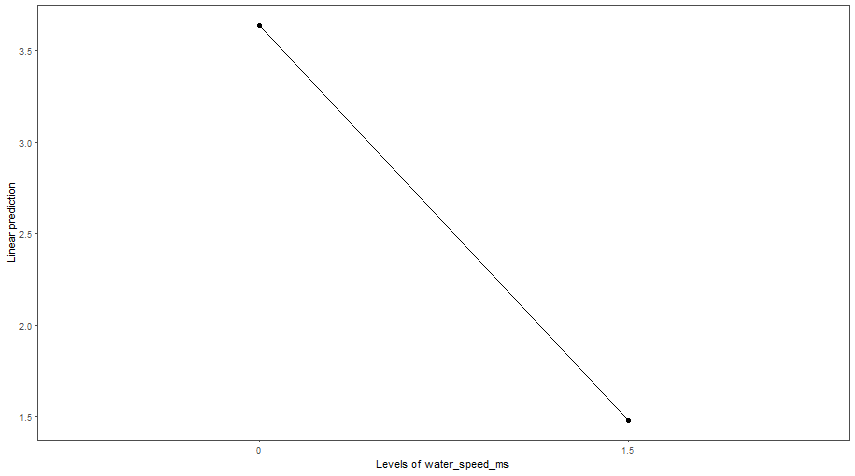


**Additional file 1: Figure S3**.Estimated slope of *Bulinus* spp with water speed.

**Additional file 1: Table S2:** *B. truncatus* glmmTMB negative binomial model summary output

| BT_m1 | coefs | Estimate | Std..Error | z.value | Pr...z.. |
| --- | --- | --- | --- | --- | --- |
| 1 | (Intercept) | -0.252 | 1.169 | -0.215 | 0.830 |
| 2 | wmo_prec | -0.058 | 0.097 | -0.601 | 0.548 |
| 3 | site_typecanal.3 | -0.860 | 0.308 | -2.789 | 0.005 |
| 4 | site_typepond | -0.078 | 0.600 | -0.130 | 0.896 |
| 5 | site_typerice.p | -1.915 | 0.487 | -3.932 | 0.000 |
| 6 | site_typeriver | 0.125 | 0.610 | 0.205 | 0.837 |
| 7 | site_typerivulet | -0.611 | 0.840 | -0.728 | 0.467 |
| 8 | site_typespillway | -3.182 | 0.841 | -3.783 | 0.000 |
| 9 | month2 | 0.841 | 0.380 | 2.216 | 0.027 |
| 10 | month3 | 1.462 | 0.376 | 3.887 | 0.000 |
| 11 | month4 | 1.618 | 0.552 | 2.933 | 0.003 |
| 12 | month5 | 0.837 | 0.382 | 2.190 | 0.029 |
| 13 | month6 | 0.379 | 0.372 | 1.019 | 0.308 |
| 14 | month7 | -0.339 | 0.367 | -0.922 | 0.356 |
| 15 | month8 | -1.010 | 0.410 | -2.464 | 0.014 |
| 16 | month9 | -0.496 | 0.373 | -1.329 | 0.184 |
| 17 | month10 | -0.602 | 0.356 | -1.691 | 0.091 |
| 18 | month11 | -0.444 | 0.367 | -1.211 | 0.226 |
| 19 | month12 | -0.334 | 0.353 | -0.944 | 0.345 |
| 20 | BT_pos_tot | 0.414 | 0.071 | 5.811 | 0.000 |
| 21 | localityDiambala | 0.402 | 1.208 | 0.333 | 0.739 |
| 22 | localityDoguel Kaina | -1.834 | 1.097 | -1.672 | 0.094 |
| 23 | localityDokimana | -2.294 | 1.498 | -1.531 | 0.126 |
| 24 | localityKarma | -4.313 | 1.261 | -3.420 | 0.001 |
| 25 | localityKohan Garantche | -1.492 | 1.534 | -0.973 | 0.331 |
| 26 | localityKoutoukale Zeno | -0.646 | 1.234 | -0.523 | 0.601 |
| 27 | localityLata Kabia | -0.886 | 1.184 | -0.748 | 0.454 |
| 28 | localityLibore | -0.900 | 1.182 | -0.761 | 0.447 |
| 29 | localityNamari Goungou | 1.073 | 1.220 | 0.880 | 0.379 |
| 30 | localityNamaro | -1.851 | 1.221 | -1.515 | 0.130 |
| 31 | localitySay | -1.530 | 1.281 | -1.195 | 0.232 |
| 32 | localitySeberi | 0.411 | 1.215 | 0.338 | 0.735 |
| 33 | localityTagabati | -2.634 | 1.143 | -2.305 | 0.021 |
| 34 | localityTiaguirire | -1.420 | 1.306 | -1.087 | 0.277 |
| 35 | localityTokoye | -2.462 | 1.270 | -1.938 | 0.053 |
| 36 | localityYoreize Koira | -2.890 | 1.253 | -2.307 | 0.021 |
| 37 | localityYouri | -1.215 | 1.109 | -1.095 | 0.273 |
| 38 | localityZama Koira Tegui | -3.623 | 1.231 | -2.943 | 0.003 |
| 39 |  |  |  |  |  |

**Additional file 1: Table S3:** *B. forskalii* glmmTMB negative binomial model summary output

| BF_m1 | coefs | Estimate | Std..Error | z.value | Pr...z.. |
| --- | --- | --- | --- | --- | --- |
| 1 | (Intercept) | -2.193 | 1.125 | -1.950 | 0.051 |
| 2 | wmo_prec | -0.075 | 0.095 | -0.793 | 0.427 |
| 3 | site_typecanal.3 | 0.800 | 0.283 | 2.828 | 0.005 |
| 4 | site_typepond | 0.445 | 0.520 | 0.856 | 0.392 |
| 5 | site_typerice.p | 0.352 | 0.420 | 0.839 | 0.402 |
| 6 | site_typeriver | -1.819 | 0.584 | -3.115 | 0.002 |
| 7 | site_typerivulet | -1.887 | 0.803 | -2.349 | 0.019 |
| 8 | site_typespillway | 0.963 | 0.663 | 1.453 | 0.146 |
| 9 | month2 | 0.786 | 0.407 | 1.934 | 0.053 |
| 10 | month3 | 1.171 | 0.403 | 2.902 | 0.004 |
| 11 | month4 | 1.582 | 0.582 | 2.718 | 0.007 |
| 12 | month5 | 0.750 | 0.404 | 1.856 | 0.063 |
| 13 | month6 | 0.560 | 0.397 | 1.410 | 0.159 |
| 14 | month7 | -0.068 | 0.393 | -0.172 | 0.864 |
| 15 | month8 | 0.560 | 0.427 | 1.313 | 0.189 |
| 16 | month9 | 1.362 | 0.400 | 3.405 | 0.001 |
| 17 | month10 | 0.206 | 0.381 | 0.541 | 0.589 |
| 18 | month11 | 0.227 | 0.391 | 0.580 | 0.562 |
| 19 | month12 | 0.237 | 0.377 | 0.629 | 0.529 |
| 20 | localityDiambala | -1.402 | 1.149 | -1.220 | 0.223 |
| 21 | localityDoguel Kaina | -0.254 | 1.046 | -0.243 | 0.808 |
| 22 | localityDokimana | -0.670 | 1.384 | -0.484 | 0.628 |
| 23 | localityGantchi Bassarou | -2.238 | 1.308 | -1.711 | 0.087 |
| 24 | localityKarma | -1.025 | 1.162 | -0.882 | 0.378 |
| 25 | localityKohan Garantche | 0.986 | 1.443 | 0.683 | 0.495 |
| 26 | localityKoutoukale Zeno | -0.207 | 1.172 | -0.177 | 0.860 |
| 27 | localityLata Kabia | 0.262 | 1.124 | 0.233 | 0.816 |
| 28 | localityLibore | -0.817 | 1.127 | -0.725 | 0.468 |
| 29 | localityNamari Goungou | -1.093 | 1.158 | -0.944 | 0.345 |
| 30 | localityNamaro | -0.770 | 1.158 | -0.664 | 0.506 |
| 31 | localitySay | -1.136 | 1.246 | -0.911 | 0.362 |
| 32 | localitySeberi | 0.053 | 1.152 | 0.046 | 0.963 |
| 33 | localityTagabati | -1.112 | 1.110 | -1.001 | 0.317 |
| 34 | localityTiaguirire | -1.465 | 1.236 | -1.186 | 0.236 |
| 35 | localityTokoye | -0.843 | 1.201 | -0.702 | 0.483 |
| 36 | localityYoreize Koira | -1.668 | 1.185 | -1.408 | 0.159 |
| 37 | localityYouri | -0.291 | 1.060 | -0.274 | 0.784 |
| 38 | localityZama Koira Tegui | -1.636 | 1.157 | -1.414 | 0.157 |
| 39 | BF_pos_tot | 0.537 | 0.345 | 1.558 | 0.119 |

**Additional file 1: Table S4:** *Radix natalensis* glmmTMB negative binomial model summary output

| RN_m1 | coefs | Estimate | Std..Error | z.value | Pr...z.. |
| --- | --- | --- | --- | --- | --- |
| 1 | (Intercept) | -8.221 | 2.104 | -3.906 | 0.000 |
| 2 | wmo_prec | 0.056 | 0.206 | 0.270 | 0.787 |
| 3 | site_typecanal.3 | 0.396 | 0.702 | 0.564 | 0.573 |
| 4 | site_typepond | 4.215 | 1.257 | 3.354 | 0.001 |
| 5 | site_typerice.p | 2.022 | 0.883 | 2.290 | 0.022 |
| 6 | site_typeriver | 3.109 | 1.287 | 2.416 | 0.016 |
| 7 | site_typerivulet | 3.157 | 1.570 | 2.010 | 0.044 |
| 8 | site_typespillway | 0.434 | 1.877 | 0.231 | 0.817 |
| 9 | month2 | 1.586 | 0.617 | 2.570 | 0.010 |
| 10 | month3 | 2.734 | 0.610 | 4.485 | 0.000 |
| 11 | month4 | 2.794 | 0.873 | 3.201 | 0.001 |
| 12 | month5 | 0.748 | 0.646 | 1.159 | 0.246 |
| 13 | month6 | 0.463 | 0.674 | 0.686 | 0.493 |
| 14 | month7 | -1.780 | 0.735 | -2.421 | 0.015 |
| 15 | month8 | -1.902 | 0.795 | -2.392 | 0.017 |
| 16 | month9 | -2.043 | 0.761 | -2.683 | 0.007 |
| 17 | month10 | -2.019 | 0.735 | -2.748 | 0.006 |
| 18 | month11 | -1.161 | 0.713 | -1.628 | 0.104 |
| 19 | month12 | -0.656 | 0.619 | -1.059 | 0.290 |
| 20 | localityDiambala | -0.556 | 2.185 | -0.255 | 0.799 |
| 21 | localityDoguel Kaina | 2.109 | 1.898 | 1.111 | 0.266 |
| 22 | localityDokimana | 0.984 | 2.594 | 0.379 | 0.704 |
| 23 | localityGantchi Bassarou | 0.478 | 2.417 | 0.198 | 0.843 |
| 24 | localityKarma | -1.366 | 2.212 | -0.617 | 0.537 |
| 25 | localityKohan Garantche | -0.434 | 2.667 | -0.163 | 0.871 |
| 26 | localityKoutoukale Zeno | -17.933 | 1414.264 | -0.013 | 0.990 |
| 27 | localityLata Kabia | -2.187 | 2.229 | -0.981 | 0.326 |
| 28 | localityLibore | -0.756 | 2.129 | -0.355 | 0.722 |
| 29 | localityNamari Goungou | 2.697 | 2.158 | 1.250 | 0.211 |
| 30 | localityNamaro | -2.226 | 2.291 | -0.972 | 0.331 |
| 31 | localitySay | -2.602 | 2.594 | -1.003 | 0.316 |
| 32 | localitySeberi | -2.291 | 2.402 | -0.954 | 0.340 |
| 33 | localityTagabati | -0.647 | 1.944 | -0.333 | 0.739 |
| 34 | localityTiaguirire | 2.032 | 2.279 | 0.892 | 0.372 |
| 35 | localityTokoye | 3.591 | 2.207 | 1.627 | 0.104 |
| 36 | localityYoreize Koira | 2.250 | 2.173 | 1.036 | 0.300 |
| 37 | localityYouri | -2.059 | 1.941 | -1.061 | 0.289 |
| 38 | localityZama Koira Tegui | 0.295 | 2.189 | 0.135 | 0.893 |

**Additional file 1: Table S5:** *Biomphalaria pfeifferi* glmmTMB negative binomial model summary output

| bp_m1 | coefs | Estimate | Std..Error | z.value | Pr...z.. |
| --- | --- | --- | --- | --- | --- |
| 1 | (Intercept) | -5.556 | 1.326 | -4.191 | 0.000 |
| 2 | wmo_prec | -0.266 | 0.420 | -0.632 | 0.527 |
| 3 | site_typecanal.3 | -1.863 | 1.060 | -1.758 | 0.079 |
| 4 | month2 | 2.228 | 1.332 | 1.673 | 0.094 |
| 5 | month3 | 2.682 | 1.329 | 2.018 | 0.044 |
| 6 | month4 | 5.088 | 2.064 | 2.466 | 0.014 |
| 7 | month5 | 2.202 | 1.336 | 1.648 | 0.099 |
| 8 | month6 | 2.502 | 1.246 | 2.007 | 0.045 |
| 9 | month7 | -1.005 | 1.451 | -0.692 | 0.489 |
| 10 | month8 | 0.246 | 1.650 | 0.149 | 0.881 |
| 11 | month9 | 0.884 | 1.301 | 0.680 | 0.497 |
| 12 | month10 | 0.526 | 1.373 | 0.383 | 0.702 |
| 13 | month11 | -0.393 | 1.330 | -0.295 | 0.768 |
| 14 | month12 | 1.272 | 1.362 | 0.934 | 0.350 |
| 15 | localityNamari Goungou | 1.802 | 1.048 | 1.719 | 0.086 |
| 16 | BP_pos_tot | 0.517 | 0.118 | 4.372 | 0.000 |

**Additional file 1: Table S6:** *Bulinus* spp glmmTMB negative binomial model summary output

| Bulinus_m1 | coefs | Estimate | Std..Error | z.value | Pr...z.. |
| --- | --- | --- | --- | --- | --- |
| 1 | (Intercept) | 0.643 | 1.136 | 0.566 | 0.571 |
| 2 | Temp_Water | -0.211 | 0.654 | -0.323 | 0.747 |
| 3 | pH | 0.038 | 0.071 | 0.537 | 0.591 |
| 4 | water_speed_ms | -1.011 | 0.265 | -3.819 | 0.000 |
| 5 | water_depth | -0.631 | 0.244 | -2.583 | 0.010 |
| 6 | Cond | -0.005 | 0.165 | -0.030 | 0.976 |
| 7 | wmo_prec | -0.159 | 0.099 | -1.604 | 0.109 |
| 8 | site_typecanal.3 | 0.690 | 1.027 | 0.672 | 0.502 |
| 9 | site_typepond | -2.563 | 1.795 | -1.428 | 0.153 |
| 10 | site_typerice.p | 0.873 | 1.966 | 0.444 | 0.657 |
| 11 | site_typeriver | 2.358 | 1.682 | 1.402 | 0.161 |
| 12 | site_typerivulet | 5.000 | 2.389 | 2.093 | 0.036 |
| 13 | site_typespillway | -1.709 | 2.046 | -0.835 | 0.404 |
| 14 | Bulinus_pos_tot | 0.304 | 0.055 | 5.528 | 0.000 |
| 15 | month2 | 0.751 | 0.343 | 2.191 | 0.028 |
| 16 | month3 | 1.254 | 0.347 | 3.611 | 0.000 |
| 17 | month4 | 1.518 | 0.501 | 3.032 | 0.002 |
| 18 | month5 | 0.784 | 0.377 | 2.079 | 0.038 |
| 19 | month6 | 0.500 | 0.366 | 1.363 | 0.173 |
| 20 | month7 | 0.077 | 0.357 | 0.215 | 0.830 |
| 21 | month8 | 0.107 | 0.386 | 0.276 | 0.783 |
| 22 | month9 | 0.163 | 0.374 | 0.434 | 0.664 |
| 23 | month10 | -0.238 | 0.373 | -0.640 | 0.522 |
| 24 | month11 | 0.527 | 0.389 | 1.355 | 0.175 |
| 25 | month12 | -0.009 | 0.345 | -0.025 | 0.980 |
| 26 | localityDiambala | -0.619 | 0.843 | -0.734 | 0.463 |
| 27 | localityDoguel Kaina | -1.968 | 0.773 | -2.546 | 0.011 |
| 28 | localityDokimana | -3.046 | 1.041 | -2.926 | 0.003 |
| 29 | localityGantchi Bassarou | -3.457 | 0.984 | -3.512 | 0.000 |
| 30 | localityKarma | -2.125 | 0.859 | -2.473 | 0.013 |
| 31 | localityKohan Garantche | -2.083 | 1.064 | -1.958 | 0.050 |
| 32 | localityKoutoukale Zeno | -0.865 | 0.862 | -1.003 | 0.316 |
| 33 | localityLata Kabia | -0.836 | 0.822 | -1.018 | 0.309 |
| 34 | localityLibore | -1.637 | 0.832 | -1.968 | 0.049 |
| 35 | localityNamari Goungou | -0.059 | 0.851 | -0.070 | 0.945 |
| 36 | localityNamaro | -1.283 | 0.850 | -1.509 | 0.131 |
| 37 | localitySay | -1.807 | 0.893 | -2.023 | 0.043 |
| 38 | localitySeberi | -0.635 | 0.847 | -0.750 | 0.453 |
| 39 | localityTagabati | -1.980 | 0.818 | -2.420 | 0.016 |
| 40 | localityTiaguirire | -2.283 | 0.914 | -2.497 | 0.013 |
| 41 | localityTokoye | -2.418 | 0.887 | -2.726 | 0.006 |
| 42 | localityYoreize Koira | -2.418 | 0.868 | -2.786 | 0.005 |
| 43 | localityYouri | -1.757 | 0.778 | -2.259 | 0.024 |
| 44 | localityZama Koira Tegui | -2.849 | 0.851 | -3.349 | 0.001 |
| 45 | Temp_Water:site_typecanal.3 | 0.018 | 0.687 | 0.027 | 0.979 |
| 46 | Temp_Water:site_typepond | 1.716 | 1.093 | 1.570 | 0.117 |
| 47 | Temp_Water:site_typerice.p | -1.537 | 1.227 | -1.252 | 0.211 |
| 48 | Temp_Water:site_typeriver | -4.713 | 1.262 | -3.735 | 0.000 |
| 49 | Temp_Water:site_typerivulet | -2.924 | 1.511 | -1.935 | 0.053 |
| 50 | Temp_Water:site_typespillway | 3.990 | 1.646 | 2.424 | 0.015 |
| 51 | pH:site_typecanal.3 | -0.120 | 0.087 | -1.376 | 0.169 |
| 52 | pH:site_typepond | 0.001 | 0.155 | 0.009 | 0.992 |
| 53 | pH:site_typerice.p | -0.080 | 0.172 | -0.467 | 0.641 |
| 54 | pH:site_typeriver | 0.290 | 0.167 | 1.735 | 0.083 |
| 55 | pH:site_typerivulet | -0.291 | 0.174 | -1.666 | 0.096 |
| 56 | pH:site_typespillway | -0.418 | 0.212 | -1.971 | 0.049 |
| 57 | Cond:site_typecanal.3 | 0.009 | 0.233 | 0.040 | 0.968 |
| 58 | Cond:site_typepond | 0.640 | 0.246 | 2.600 | 0.009 |
| 59 | Cond:site_typerice.p | 0.579 | 0.352 | 1.645 | 0.100 |
| 60 | Cond:site_typeriver | -1.237 | 0.691 | -1.790 | 0.073 |
| 61 | Cond:site_typerivulet | -2.527 | 0.995 | -2.540 | 0.011 |
| 62 | Cond:site_typespillway | 0.320 | 0.320 | 0.998 | 0.318 |
| 63 | wmo_prec:site_typecanal.3 | -0.021 | 0.123 | -0.172 | 0.864 |
| 64 | wmo_prec:site_typepond | 0.404 | 0.180 | 2.248 | 0.025 |
| 65 | wmo_prec:site_typerice.p | 0.520 | 0.221 | 2.357 | 0.018 |
| 66 | wmo_prec:site_typeriver | -0.021 | 0.215 | -0.097 | 0.923 |
| 67 | wmo_prec:site_typerivulet | -0.419 | 0.231 | -1.816 | 0.069 |
| 68 | wmo_prec:site_typespillway | 0.226 | 0.169 | 1.338 | 0.181 |

**Additional file 1: Table S7.** Water chemistry and physical variable data, including WMO weather station data, A: averaged by month, and B: by site type (final year of ground collected data missing due to equipment failure)

| A: month | av.pH | av.cond | air.temp | wat.temp | av.prec | av.wmo.avT | av.wmo.minT | av.wmo.maxT |
| --- | --- | --- | --- | --- | --- | --- | --- | --- |
| 1 | 7.6 | 91.6 | 29.0 | 22.0 | 0.0 | 25.2 | 17.7 | 32.8 |
| 2 | 7.8 | 105.9 | 29.2 | 23.4 | 0.0 | 28.3 | 20.4 | 36.2 |
| 3 | 7.9 | 105.7 | 32.0 | 26.3 | 0.0 | 32.6 | 25.8 | 39.3 |
| 4 | 8.3 | 107.7 | 35.6 | 30.8 | 0.1 | 35.2 | 29.1 | 41.3 |
| 5 | 7.4 | 125.8 | 33.5 | 29.8 | 1.0 | 34.6 | 28.8 | 40.5 |
| 6 | 7.1 | 147.9 | 31.0 | 28.7 | 1.3 | 32.5 | 27.4 | 37.6 |
| 7 | 7.5 | 138.5 | 29.9 | 28.0 | 6.1 | 30.4 | 25.9 | 35.0 |
| 8 | 8.0 | 119.1 | 29.9 | 28.7 | 12.5 | 28.4 | 24.8 | 32.0 |
| 9 | 7.3 | 104.0 | 31.8 | 29.7 | 1.6 | 29.9 | 25.0 | 34.8 |
| 10 | 7.9 | 87.8 | 32.5 | 29.2 | 0.0 | 31.7 | 25.4 | 38.1 |
| 11 | 8.0 | 83.8 | 31.3 | 26.3 | 0.0 | 29.4 | 21.2 | 37.5 |
| 12 | 8.4 | 93.4 | 29.0 | 23.3 | 0.0 | 25.0 | 17.2 | 32.8 |

| 2eeeeeeeeee | av.pH | av.TDS | av.cond | air.temp | water.temp | |  |  |  |
| --- | --- | --- | --- | --- | --- | --- | --- | --- | --- |
| rivulet | 7.9 | 35.0 | 72.0 | 29.8 | 26.6 |  |  |  |  |
| river | 7.7 | 35.7 | 74.7 | 31.9 | 27.7 |  |  |  |  |
| can2 | 7.7 | 49.5 | 105.2 | 30.7 | 26.7 |  |  |  |  |
| canal.3 | 7.7 | 50.1 | 106.8 | 30.3 | 26.4 |  |  |  |  |
| rice.p | 7.6 | 59.7 | 122.5 | 31.3 | 27.5 |  |  |  |  |
| pond | 7.9 | 80.9 | 164.8 | 32.3 | 28.4 |  |  |  |  |
| spillway | 7.7 | 91.5 | 200.7 | 32.6 | 27.8 |  |  |  |  |

(Summary outputs for prevalence and abundance of infected snails, and pairwise emmeans tables available on request)
